# Neural SHAKE: geometric constraints in neural differential equations

**DOI:** 10.1186/s13321-025-01053-w

**Published:** 2025-08-04

**Authors:** Justin S. Diamond, Markus A. Lill

**Affiliations:** Department of Pharmaceutical Sciences, University of Basel, Basel, Switzerland

**Keywords:** Diffusion, Constraints, Molecular Generation

## Abstract

**Abstract:**

Generating accurate molecular conformations hinges on sampling effectively from a high-dimensional space of atomic arrangements, which grows exponentially with system size. To ensure physically valid geometries and increase the likelihood of reaching low-energy conformations, it is us ful to incorporate prior physicsbased information by recasting them as geometric constraints that naturally arise as nonlinear constraint satisfaction problems. In this work, we propose an approach to embed these strict constraints into neural differential equations, leveraging the denoising diffusion framework. By projecting the stochastic generative dynamics onto a manifold defined by constraint sets, our method enforces exact feasibility at each step, unlike alternative approaches that merely impose soft constraints through probabilistic guidance. This technique generates lower-energy molecular conformations, enables more efficient subspace exploration, and formally subsumes classifier-guidance-type methods by treating geometric constraints as strict algebraic conditions within the diffusion process.

**Scientific Contribution:**

Neural SHAKE formulates exact manifold‑projected score‑based diffusion : each reverse-SDEincrement is orthogonally projected, via a Lagrange-multiplier solve, onto the constraint surfaceσₐ(x)=0 for a = 1,…, A, with A the number of independent constraints and thus the manifold’scodimension . This projection preserves global SE(3) symmetry and enforces constraints tosolver tolerance. It induces a well-posed surface Fokker–Planck flow on the (3 N − A)-dimensional manifold, while a coarea/Fixman Jacobian carries the ambient 3 N-dimensionaldensity to a normalized density on that manifold, preserving probability mass after the dimensionality reduction.

## Introduction

Sampling accurate molecular conformations [1, 2] is a critical challenge in computational chemistry. The space of possible atomic arrangements increases exponentially with the degrees of freedom in the system. To manage this complexity, prior information about geometric arrangements, such as bond lengths, bond angles, and dihedral angles, is crucial. These geometric constraints help in generating physically realistic structures that are necessary for various applications, including drug discovery and materials science.

Geometric constraints are essential because they reduce the vast conformational space to a more manageable subset that is physically plausible [3]. For example, consider the case where we have a ligand with a known binding pocket [4]. Instead of performing sampling over the entire protein, we can enforce the ligand to be constrained to a physical volume defining the binding pocket. One can also use constraints to sample low-energy conformations of molecules that are more likely to come from the desired Boltzmann distribution. To see how to use constraints to sample more likely molecular conformations we can look at how Propagators are defined in Molecular Dyanmics.

Molecular Dynamics (MD) parameterizes the motion of molecular systems using a sum of harmonic, trigonometric, and inverse power functions that approximate the quantum potential energy surface of a particular molecule. The equations of motion (EOM) for a molecular system can be derived from the Lagrangian, incorporating both the kinetic energy $T$ and the potential energy $U$, as well as geometric constraints $\sigma _a$:

$$\begin{aligned} L = T - U = \frac{1}{2} M \left( \frac{d x}{d t}\right) ^2 - U \end{aligned}$$

The equations of motion for a constrained system in classical (Newtonian) Molecular Dynamics (MD) are given by:

1 $$\begin{aligned} M \frac{d^2 x}{d t^2} = -\nabla U - \sum _a \lambda _a \nabla \sigma _a, \end{aligned}$$

where $M$ is the mass matrix, $x$ represents Cartesian coordinates, $t$ is time, $\lambda _a$ are Lagrange multipliers, and $\sigma _a$ are holonomic constraints on the system. This follows from the application of Lagrange multipliers in classical mechanics, ensuring that the time evolution remains consistent with the constraint surfaces [5].

However, our interest lies in adapting these constraint-enforcement principles to a diffusion-based setting, which naturally corresponds to an overdamped or Brownian regime rather than the full Newtonian dynamics of Equation (1). In the overdamped limit, the inertial term $M \frac{d^2 x}{dt^2}$ becomes negligible, and the time evolution is governed primarily by frictional and stochastic forces. Mathematically, this is often written as:

2 $$\begin{aligned} \gamma \frac{dx}{dt} = -\nabla U - \sum _a \lambda _a \nabla \sigma _a + \text {stochastic noise}, \end{aligned}$$

where $\gamma$ is a friction (drag) coefficient.

### Transitioning to neural diffusion processes

To generalize these concepts into neural diffusion processes, we translate the idea of constraint projection–familiar from Molecular Dynamics–into the Stochastic Differential Equation (SDE) framework used for denoising-based generative models [6, 7]. Specifically, rather than evolving physical coordinates in real time with inertial dynamics, we evolve latent or molecular coordinates stochastically from a simple prior distribution to the target distribution of molecular conformations. Throughout this evolution, we project each stochastic update onto the subspace that satisfies the desired geometric constraints.

**Fig. 1:**
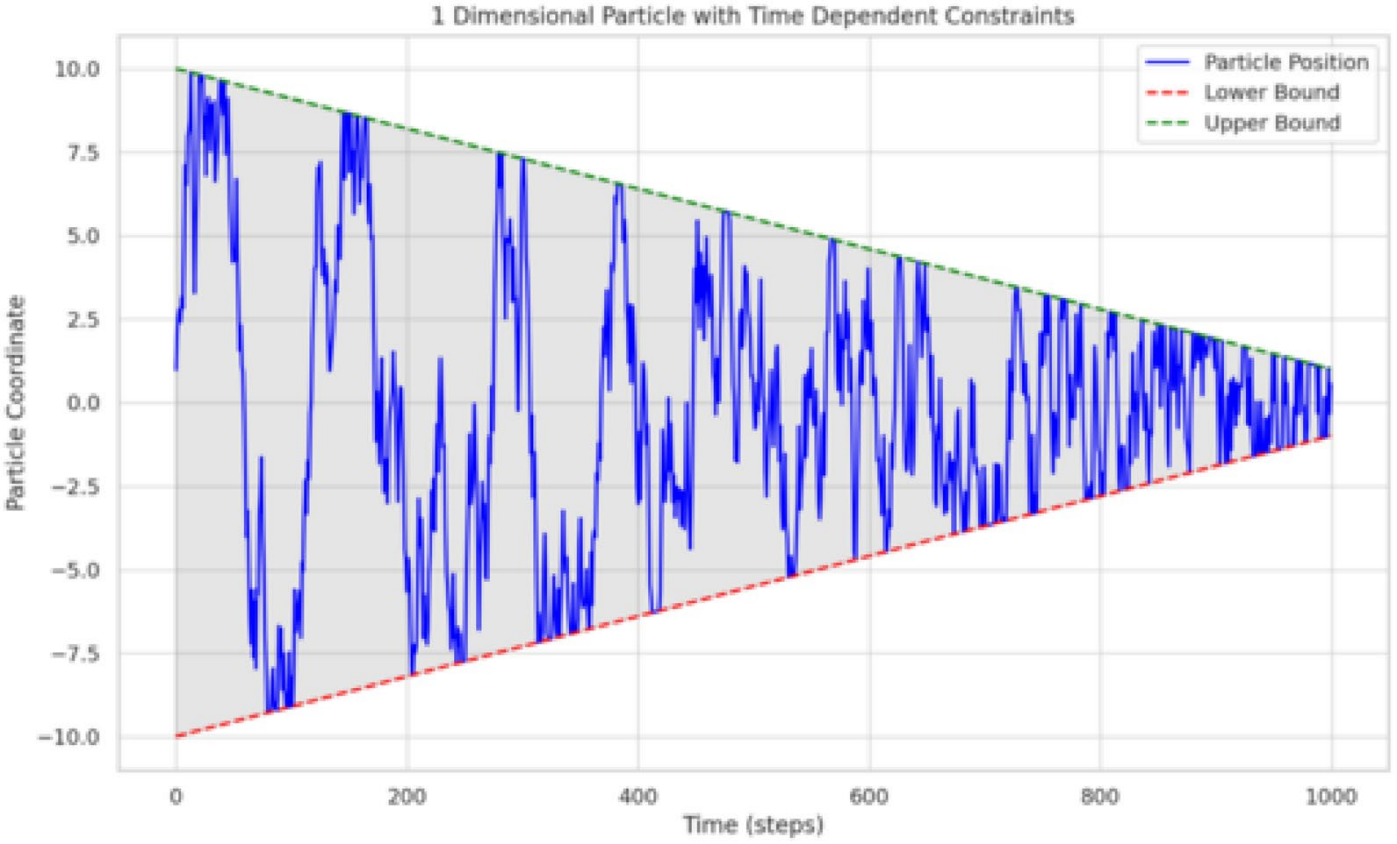
Illustrative example of random motion of a 1D particle with time-dependent constraints over 1000 steps

Formally, consider a constraint on the distance between atoms $i$ and $j$, given by $\sigma _{d_{ij}} = (d_{ij} - d_{ij,0})^2 = 1.5$. We incorporate this constraint into a diffusion process by projecting out noise components that would violate $\sigma _{d_{ij}}$. Concretely, the SDE includes a projection term $P$ onto the null-space of $\nabla \sigma _{d_{ij}}$:

3 $$\begin{aligned} dx = \sqrt{2D} \underbrace{\left( I - \frac{\nabla \sigma _{d_{ij}} \nabla \sigma _{d_{ij}}^T}{\Vert \nabla \sigma _{d_{ij}}\Vert ^2}\right) }_{\displaystyle P} dB - D \nabla \log p_t(x) dt, \end{aligned}$$

where $D$ is a diffusion constant, $B$ is standard Brownian motion, and $\nabla \sigma _{d_{ij}}$ is the gradient of the constraint function. The operator $P$ removes any noise component incompatible with the constraint, ensuring the particle does not stray from the constraint-satisfying manifold. Thus, we obtain a modified diffusion process on geometrically constrained subspaces, paralleling the role of $\lambda _a \nabla \sigma _a$ in Equation (1), but under an overdamped/stochastic regime Fig. 1.

This projection technique allows us to embed molecular constraints into neural SDEs, just as one would enforce constraints in classical dynamics–yet now we do so in the context of denoising diffusion for generative modeling of molecular conformations. Whereas the prior is used primarily to speed up integration schemes, the latter is used for a fundamentally different problem, sampling low energy subspaces of molecular conformations.

### Related work

Neural SHAKE has been designed to handle geometric constraints in graph generative models by leveraging Lagrange multipliers and iterative corrections. This section reviews other significant approaches in the field that address similar constraint satisfaction problems.

There are two primary subgroups of related work: the incorporation of constraints into molecular generation tasks and the incorporation of constraints into generative diffusion processes.

For molecular generation, [8] introduced scaffold-based generation to ensure that molecules satisfy specific scaffold descriptions of branching covalent bond networks, using reinforcement learning to guide the process. Similarly, [9] utilized reinforcement learning to distill a generative model tailored to specific chemical domains. These methods rely on empirical definitions of constraints related to molecular generation while training models to satisfy these constraints.

In contrast, [10, 11, 12] developed theoretical frameworks for incorporating constraints into diffusion processes. Reflected diffusion models address constraints in stochastic processes they They also presented an approach using Metropolis sampling for constrained diffusion models, which approximates barrier methods by adding a potential that becomes infinite as the diffusion process encounters barriers.

An alternative route to geometrically valid sampling is to parameterise the molecule directly in internal coordinates–that is, bond lengths, bond angles, and dihedral angles–thereby avoiding specific types of Cartesian-space constraint violations altogether. Internal–coordinate kinematics has a long history in computational chemistry. Analytical loop-closure techniques were first used to enforce exact distance/angle constraints; most notably, Coutsias *et al.* derived closed-form solutions for protein loop closure with fixed bond lengths and angles, obtaining up to sixteen feasible conformations for six independent torsional degrees of freedom [13]. A subsequent paper introduced resultant-based algorithms to enumerate *all* conformations that satisfy a given nonlinear constraint set [14].

Recently, machine-learning approaches have begun exploiting internal coordinates as well. Torsional diffusion models operate directly on the hypertorus of molecular dihedral angles. Jing *et al.* [15] formulate a score-based diffusion process on that space. Similarly, DiffPack diffuses protein side-chain torsions autoregressively to maintain stereochemical validity throughout generation [16]. In the protein-loop context, O’Donnell *et al.* have proposed a global backbone-angle parameterisation to enhance conformational exploration [17], building on their earlier geometric analysis of tripeptide constraints [18].

Neural SHAKE generalizes the approach of [19] to work with diffusion processes by incorporating time-dependent constraints to interpolate between a prior Gaussian distribution and a possibly disjoint solution space of molecules that satisfy the constraints using Lagrange multipliers. Furthermore, our method can be applied modularly without additional training, whereas existing approaches rely on incorporating constraints into the training scheme. It is a strict generalization of the above cases, as we can also specify internal geometric constraints to be satisfied at the cost of additional complexities.

In addition to diffusion models, many established methods exist for exploring molecular conformations. Basin-hopping global optimization is a popular approach that iteratively perturbs structures and then locally minimizes the energy. Wales’s energy landscapes framework  [20] provides the theoretical foundation for such methods, treating the conformational search as finding minima on a rugged potential energy surface. Techniques like Minima Hopping and random torsion twisting are widely used to find low-energy conformers. For example, Roth et al. [21] combined basin hopping with rapidly exploring random trees to more efficiently navigate high-dimensional energy surfaces, successfully identifying diverse low-energy states in a model protein. Furthermore, enhanced sampling algorithms targeting thermodynamic ensembles (e.g., umbrella sampling, metadynamics, and related free-energy computation methods) have been developed in the chemistry literature. We now cite the monograph by Lelièvre et al. 22] which covers such techniques for obtaining Boltzmann-weighted ensembles. We emphasize that Neural SHAKE serves a complementary role: it is a generative sampling approach that can quickly propose plausible conformers that satisfy user given or generated sets of constraints.

### Contributions

Our methodology bridges the gap between classical mechanics and modern generative models, providing a framework for constrained molecular sampling of low-energy conformations, where constraints from classical propogators (or other priors) define permissible geometric subspaces that molecules must adhere to.

1. We incorporate a generalized SHAKE algorithm, called Neural SHAKE, into diffusion processes to enforce nonlinear constraint sets, such as bond lengths, angles, and dihedral relationships, which do not necessarily form a simple linear system.
2. Coupling Neural SDEs with constraint-induced ODEs, where the ODE arises from the constraint satisfaction problem, to integrate physical constraints directly into the generative diffusion process.
3. Generalizing classifier guidance [23] to incorporate multiple interacting constraint functions, treating constraint satisfaction as a guidance mechanism that enforces physically valid molecular conformations during diffusion.
4. Demonstrating the approach on a simple example, such as butane, to distinguish between eclipsed and staggered conformations.
5. Extending [24] by analyzing general trends on a set of molecules from DrugBank, generating sets of valid constraints with generative methods, and formalizing the theoretical foundations of Neural SHAKE.

## Methods

### Diffusion models for molecular conformer generation

#### Forward and reverse processes

Let $x \in {\mathbb {R}}^{3N}$ denote the coordinates of a molecular system. In a continuous-time diffusion framework [25, 7], one defines a forward (noising) Stochastic Differential Equation (SDE):

4 $$\begin{aligned} d x = f(x, t)\,dt + g(t)\, dW_t, \end{aligned}$$

where $W_t$ is a standard Wiener process. Over time $t \in [0,T]$, the distribution of $x$ transitions from the empirical (training) distribution $p_0(x)$ to a simple prior $\pi (x)$ (e.g. a Gaussian). A neural network is trained to approximate the time-dependent score $\nabla \log p_t(x)$. In practice, one samples from $\pi (x)$ and integrates the corresponding *reverse* SDE:

5 $$\begin{aligned} d x = \bigl [f(x,t) - g(t)^2\,\nabla \log p_t(x)\bigr ]\,dt + g(t)\, d{\widetilde{W}}_t, \end{aligned}$$

with ${\widetilde{W}}_t$ another standard Wiener process but in the reverse-time direction $t: T \rightarrow 0$. This construction yields samples from $p_0(x)$ in the limit of exact score estimation [26].

In molecular applications, $p_0(x)$ can be the distribution of valid conformers for a given molecule. The learned score $\nabla \log p_t(x)$ encodes structural regularities (e.g., typical bond lengths, angles, etc.). Generating new conformers involves solving (5) from a noise-driven initial condition to a final molecular configuration.

### Constrained molecular dynamics and the SHAKE algorithm

#### Equations of motion with constraints

Classical Molecular Dynamics (MD) evolves atomic coordinates $x \in {\mathbb {R}}^{3N}$ under a potential energy function $U(x)$. In the unconstrained, Newtonian formulation,

6 $$\begin{aligned} M \,\frac{d^2 x}{d t^2} \;=\; -\,\nabla U(x), \end{aligned}$$

where $M$ is a (diagonal) mass matrix. In practice, one often imposes *holonomic constraints* $\{\sigma _a(x) = 0\}_{a=1}^A$, typically to eliminate high-frequency bond oscillations (e.g., X–H stretches) that would otherwise necessitate sub-femtosecond integration steps. By fixing these degrees of freedom, the simulation can safely employ larger time steps, significantly accelerating exploration of relevant configurational space.

The constrained equations of motion become:

7 $$\begin{aligned} M \,\frac{d^2 x}{d t^2} \;=\; -\,\nabla U(x) -\sum _{a=1}^A \lambda _a \,\nabla \sigma _a(x), \end{aligned}$$

where $\lambda _a$ are Lagrange multipliers enforcing $\sigma _a(x) = 0$ at each time step [5]. These additional force-like terms project the atomic accelerations onto a subspace consistent with the constraints, thereby restricting undesired bond vibrations.

#### SHAKE algorithm

In a typical MD integrator (e.g., velocity Verlet), unconstrained *trial* coordinates ${\tilde{x}}$ obtained from a time step may violate $\sigma _a({\tilde{x}}) = 0$. The SHAKE algorithm [27] corrects these trial coordinates via an iterative projection. For each atom $x_i$, one updates:

8 $$\begin{aligned} x_i \;\leftarrow \; x_i - \sum _{a=1}^A \lambda _a \, \nabla _i \sigma _a\bigl ({\tilde{x}}\bigr ), \end{aligned}$$

where $\nabla _i \sigma _a({\tilde{x}})$ is the partial gradient of the constraint $\sigma _a$ with respect to atom $x_i$. The multipliers $\{\lambda _a\}$ are determined by solving:

9 $$\begin{aligned} \sum _{b=1}^A \lambda _b \, \bigl [\, \nabla \sigma _a\bigl ({\tilde{x}}\bigr ) \cdot \nabla \sigma _b\bigl ({\tilde{x}}\bigr ) \bigr ] \;=\; -\, \sigma _a\bigl ({\tilde{x}}\bigr ), \quad a=1,\dots ,A, \end{aligned}$$

thus ensuring $\sigma _a(x)\approx 0$ within a chosen tolerance. By removing high-frequency vibrations from the solution trajectory, SHAKE allows larger integration steps (e.g., 1–2 fs rather than 0.5 fs), expediting simulations without losing relevant low-frequency dynamics. Consequently, constraints such as fixed bond lengths are standard practice in classical MD, especially for biomolecular systems containing many hydrogen atoms.

In many biomolecular simulations, inertial terms are negligible, leading to an overdamped (Langevin or Brownian) formulation:

10 $$\begin{aligned} \gamma \frac{dx}{dt} = -\,\nabla U(x) - \sum _{a=1}^A \lambda _a \,\nabla \sigma _a(x) + \sqrt{2 \gamma k_B T}\,\eta (t), \end{aligned}$$

where $\eta (t)$ is a standard noise term. Constraints are still enforced via SHAKE-like projection steps [27].

## Neural SHAKE: constrained diffusion with time-dependent holonomic constraints

**Fig. 2:**
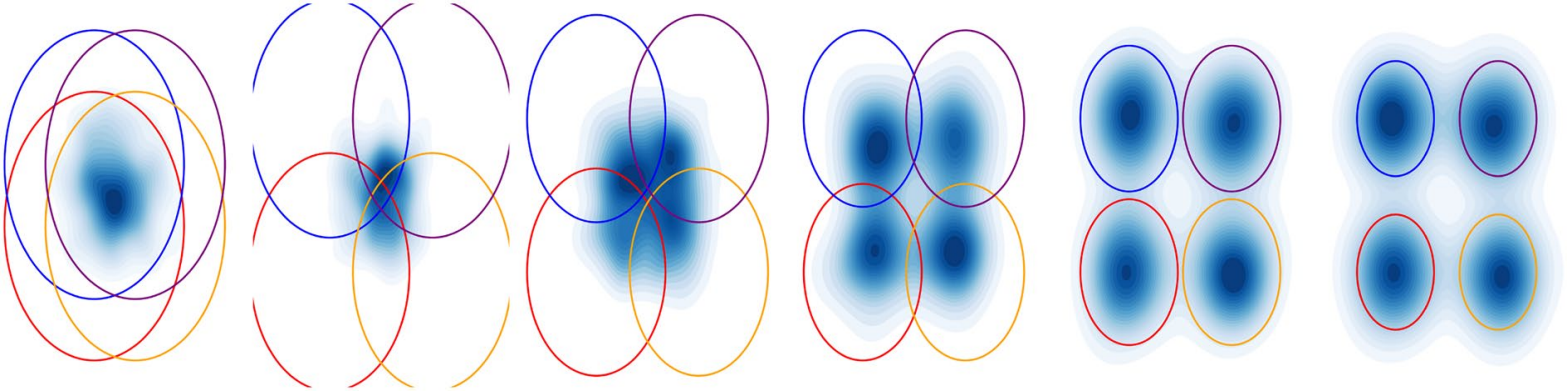
Transformation of a 2D density under evolving constraints. The circles act as constraints that continuously deform the density into the feasible region defined by the constraints at the final frame

In this section, we formally introduce the Neural SHAKE framework as a method for enforcing time-dependent holonomic constraints during diffusion processes, visualized in Figure 2. Our goal is to construct a mathematical foundation for modeling constrained diffusion dynamics while ensuring the constraints remain satisfied at each time step. We formulate the problem as a coupled set of differential equations or as a stochastic differential-algebraic equation [28], where a stochastic process governs unconstrained motion while deterministic equations enforce constraints.

We begin by establishing the formulation and defining the feasible constraint manifold. We then discuss the theoretical properties of constraint satisfaction, including manifold structure, codimension analysis, and numerical integration strategies for enforcing constraints within a diffusion process that make the process well defined. Finally, we present results on the existence and uniqueness of constrained trajectories, supported by a local rank preservation theorem that guarantees stable constraint enforcement throughout the evolution of the system. These results mostly exist in literature, but we reformulate them for our context here.

### Problem formulation

Let $x(t) \in {\mathbb {R}}^{d}$ and $\zeta (t) \in {\mathbb {R}}^{m}$, where *x* follows a stochastic process and $\zeta$ evolves deterministically. We write:

11 $$\begin{aligned} \frac{dx}{dt}&= f\bigl (t,x,\zeta \bigr )\;+\;g\bigl (t\bigr )\,\eta (t), \end{aligned}$$

12 $$\begin{aligned} \frac{d\zeta }{dt}&= h\bigl (t,\zeta \bigr ), \end{aligned}$$

where $\eta (t)$ is a *d*-dimensional white noise, and *f*, *g*, *h* are sufficiently smooth. We impose *time-dependent holonomic constraints*

13 $$\begin{aligned} \sigma _a\bigl (x(t),\,\zeta (t)\bigr ) \;=\; 0, \quad a = 1,\dots ,A. \end{aligned}$$

Thus, (11)–(13) define a *stochastic differential–algebraic equation (SDAE)*, coupling an SDE in *x* with an ODE in $\zeta$, subject to *A* algebraic constraints.

### Feasible set and codimension

For each fixed $\zeta$, define

$$\begin{aligned} {\mathcal {M}}(\zeta ) \;=\; \Bigl \{\, x \in {\mathbb {R}}^{d}\;\Big |\;\sigma _1(x,\zeta )=\cdots =\sigma _A(x,\zeta )=0 \Bigr \}. \end{aligned}$$

If the Jacobian matrix of partial derivatives

$$\begin{aligned} D_x \varSigma (x,\zeta ) \;=\; \begin{pmatrix} \nabla _x\,\sigma _1(x,\zeta )^\top \\ \vdots \\ \nabla _x\,\sigma _A(x,\zeta )^\top \end{pmatrix} \end{aligned}$$

has rank *A* at $(x,\zeta )$, then each connected component of ${\mathcal {M}}(\zeta )$ is a (local) submanifold of dimension $d - A$. In practice, $d=3N$ for a molecular system and *A* is the number of holonomic constraints.

### Discrete integration and projection

A time-stepping scheme on [0, *T*] can be written:

$$\begin{aligned} \zeta _{n+1}&= \zeta _n + h\bigl (t_n,\zeta _n\bigr )\,\varDelta t,\\ {\tilde{x}}&= x_n + f\bigl (t_n,x_n,\zeta _n\bigr )\,\varDelta t + \sqrt{\varDelta t}\,\eta ^n,\\ x_{n+1}&= \varPi _{{\mathcal {M}}(\zeta _{n+1})}({\tilde{x}}), \end{aligned}$$

where $\eta ^n\sim {\mathcal {N}}(0,I)$ is a discrete noise increment, and $\varPi _{{\mathcal {M}}(\zeta _{n+1})}$ projects ${\tilde{x}}$ onto the manifold ${\mathcal {M}}(\zeta _{n+1})$ by enforcing $\sigma _a\bigl (x_{n+1},\zeta _{n+1}\bigr )=0$.

### Existence and uniqueness of the constrained trajectory

#### Local existence

Consider a continuous path $\bigl (x(t),\zeta (t)\bigr )$ with $t \in [0,T]$ such that $\sigma _a\bigl (x(t),\zeta (t)\bigr )=0$ for all *a*. Suppose:

(i) *f*, *g*, and *h* are locally Lipschitz in $(x,\zeta )$ (for the drift/ODE parts).
(ii) Each $\sigma _a(x,\zeta )$ is ${\mathcal {C}}^1$ and the set ${\mathcal {M}}(\zeta )$ stays nonempty near the path.
(iii) $D_x\varSigma \bigl (x(t),\zeta (t)\bigr )$ has rank *A* in a neighborhood of the path, ensuring no immediate degeneracy.

Then, by standard results on stochastic differential–algebraic equations [28], one can construct a unique local solution for $(x,\zeta )$. At each discrete time step, one solves the ODE update for $\zeta$, takes a trial SDE step for *x*, and then corrects *x* onto ${\mathcal {M}}(\zeta )$. As $\varDelta t \rightarrow 0$, this defines a continuous path $(x(t),\zeta (t))$ remaining in the feasible manifold.

##### Theorem 1

(Local Rank Preservation) [29] Let $\sigma _{a}: {\mathbb {R}}^{3N}\times [0,T]\rightarrow {\mathbb {R}}$ be $C^1$ in (*x*, *t*) for $a=1,\dots ,A$. Suppose there is a point $(x_0,t_0)$ such that $\sigma _{a}(x_0,t_0) = 0$ for all *a*, and

$$\begin{aligned} \operatorname {rank}\bigl (D_x \varSigma (x_0,t_0)\bigr ) = A. \end{aligned}$$

Then there exists a neighborhood $U \subset {\mathbb {R}}^{3N} \times [0,T]$ of $(x_0,t_0)$ such that for every $(x,t)\in U$, the matrix $D_x\varSigma (x,t)$ also has rank *A*. In particular, no small perturbation of $(x_0,t_0)$ can immediately reduce the rank below *A*.

##### Proof

( Sketch) [29] Because each $\sigma _{a}$ is $C^1$, the map $(x,t)\mapsto \nabla _x \sigma _{a}(x,t)$ is continuous. Select any $A \times A$ submatrix of $D_x\varSigma (x,t)$ that is nonsingular at $(x_0,t_0)$. By continuity of determinants, a sufficiently small neighborhood *U* of $(x_0,t_0)$ will preserve the non-zero determinant of that submatrix. Hence the full rank *A* condition is maintained in *U*. $\square$

If $\operatorname {rank}(D_x \varSigma (x_0,t_0))=A$ and $\sigma _{a}(x_0,t_0) = 0$ for all *a*, then a direct application of the Implicit Function Theorem tells us that for *t* fixed near $t_0$, the set $\{x: \sigma _{a}(x,t)=0\}$ is a codimension-*A* manifold around $x_0$. By the local rank preservation result, this manifold structure persists for (*x*, *t*) in a neighborhood of $(x_0,t_0)$, so *M*(*t*) is well-defined and smoothly varying with *t* as long as one remains in that neighborhood. Then, for any initial condition $\bigl (x(0),\zeta (0)\bigr )$ lying in the feasible set, there exists a unique local solution $\bigl (x(t),\zeta (t)\bigr )$ on [0, *T*] that remains on the constraint manifold throughout its evolution. Here, uniqueness means that no other solution can satisfy the same initial condition and constraints without coinciding with $(x(t),\zeta (t))$ for as long as the rank condition holds. We note that a continuous path *x*(*t*) remains feasible by satisfying the constraint equations $\sigma _{a}(x(t),t)=0$. Differentiating in *t*, one obtains conditions of the form

$$\begin{aligned} \nabla _x \sigma _{a}(x(t),t)\cdot {\dot{x}}(t)\;+\;\frac{\partial }{\partial t}\sigma _{a}(x(t),t)=0, \end{aligned}$$

which can be viewed as a system of differential-algebraic equations. The full-rank condition ensures that the tangent vectors to *M*(*t*) span the correct subspace of ${\mathbb {R}}^{3N}$, guaranteeing that ${\dot{x}}(t)$ is determined consistently within a codimension-*A* manifold.

The solution stays within a well-defined manifold since because $D_x\varSigma (x,t)$ has rank *A* on *M*(*t*), it follows from the Implicit Function Theorem that each connected component of *M*(*t*) is a smooth submanifold of ${\mathbb {R}}^d$ of dimension $d - A$. We say that *M*(*t*) has codimension *A* because it is cut out by *A* independent equations. By continuity of determinants, any small perturbation near a point where $\operatorname {rank}(D_x\varSigma )=A$ cannot reduce that rank below *A*. Hence, the manifold structure is locally preserved, and the coupled SDE–ODE solution remains well-defined in a neighborhood of any regular feasible trajectory.

In practical applications such as molecular simulations or diffusion processes in constrained geometries, each constraint typically encodes a physically or geometrically distinct condition (for instance, a distance or angle constraint). Whenever these conditions do not become mutually redundant or singular, the gradients remain linearly independent in a neighborhood of the trajectory, preserving the manifold structure. Because $\sigma _{a}$ depends smoothly on both *x* and *t*, small changes in time will not abruptly destroy the independence of the gradients in a continuous system. Catastrophic rank loss would require multiple constraints to become degenerate simultaneously, an event that typically signals a genuine singularity rather than a minor perturbation. Thus, under reasonable smoothness and non-degeneracy assumptions, one obtains a robust theoretical foundation for describing and simulating diffusion or dynamics constrained by time-varying conditions.

### Neural SHAKE and guidance algorithm

We now describe how geometric constraints can be adapted to diffusion-based generative models algorithmically. This approach, *Neural SHAKE*, projects each diffusion step onto a manifold defined by a set of constraints. Below, without loss of generality (Appendix C), we focus on pairwise distance constraints, though the same technique extends to angles, dihedrals, or other geometric quantities.

#### Distance constraints from propogators

Classical propogators approximate the potential energy of a molecular system as a sum of lower-order terms (e.g. bonds, angles, torsions, and non-bonded interactions). For instance, a harmonic bond potential of the form

$$\begin{aligned} U_{\text {bond}}(r) = \tfrac{1}{2} \,k_r \,(r - r_0)^2 \end{aligned}$$

can be recast as a distance constraint

14 $$\begin{aligned} \sigma _{\textrm{bond}}(r) = \bigl (r - r_0\bigr )^2 = 0, \end{aligned}$$

where $r$ is the distance between two atoms, $r_0$ is the equilibrium distance, and $k_r$ is the force constant. More generally, we often wish to constrain *any* interatomic distance $d_{ij}$ to a target range $[\,d_{ij,\textrm{min}},\,d_{ij,\textrm{max}}\,]$ or fix it exactly at $d_{ij,0}$.

To accommodate bounded constraints, one can introduce a slack variable $s_{ij}$ such that

15 $$\begin{aligned} \sigma _{d_{ij}}(x) = \Bigl (\,\Vert x_i - x_j\Vert - d_{ij,0} - s_{ij}\Bigr )^2 = 0, \end{aligned}$$

subject to

16 $$\begin{aligned} -s_{ij,\textrm{max}} \;\le \; s_{ij} \;\le \; s_{ij,\textrm{max}}, \end{aligned}$$

where $\Vert x_i - x_j\Vert$ is the current distance between atoms $i$ and $j$, $d_{ij,0}$ is the nominal target distance, and $s_{ij,\textrm{max}}$ bounds how far the actual distance may deviate from $d_{ij,0}$. In this formulation, $\sigma _{d_{ij}}(x) = 0$ enforces that $d_{ij}$ remains within a permitted range. If $s_{ij,\textrm{max}}=0$, the distance is strictly fixed at $d_{ij,0}$.

To satisfy distance constraints during diffusion processes, one iteratively adjusts atomic positions to minimize deviations from the target constraints. The algorithm updates the positions $x_i$ and $x_j$ of the atoms involved in the constraint using the steps presented in Algorithm 1.

**Algorithm 1:**
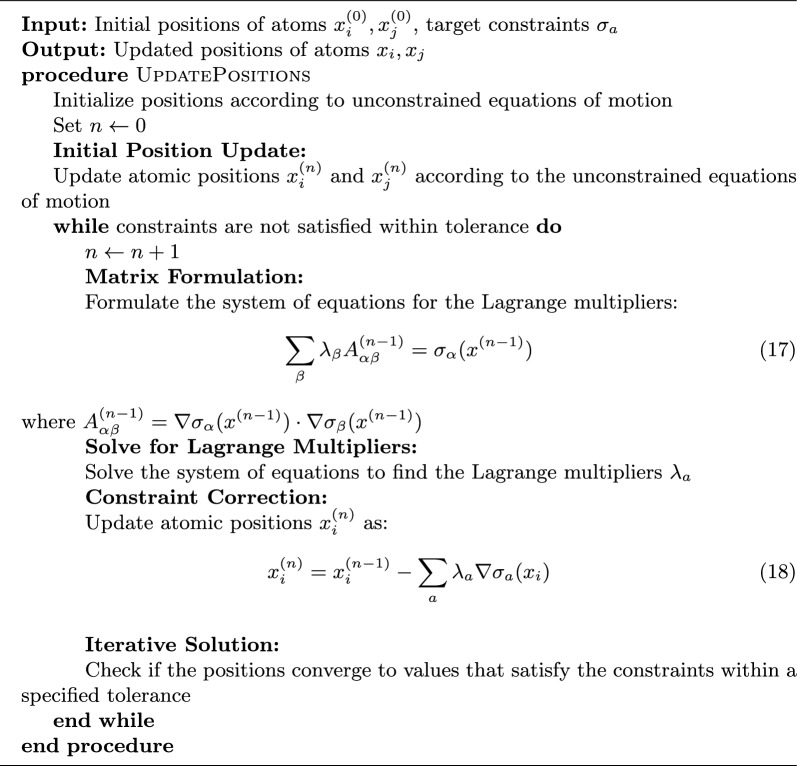
Incorporating constraints in diffusion processes

### Integrating distance restraints with a harmonic potential

### Guidance as a baseline

Guidance (Algorithm 2) methods provide a straightforward baseline by applying updates to atomic positions on a per-constraint basis, independent of other constraints. In this sense, the total update to any position $x_i$ is separated into a sum of increments, each derived from the gradient of a single constraint. For a generic constraint $\sigma _a(x)$, one can write the update step as:

19 $$\begin{aligned} x_i^{(n+1)} = x_i^{(n)} - \gamma \, \nabla \sigma _a \bigl ( x_i^{(n)} \bigr ), \end{aligned}$$

where $\gamma$ is a step size that controls the magnitude of the adjustment. Because each constraint is treated independently, no explicit mechanism exists to account for potential interactions among multiple constraints. This simplicity allows one to use Guidance as a computationally light and easily interpretable method for iteratively satisfying distance restraints, thereby serving as a natural point of comparison for more advanced methods–such as Neural Shake–that attempt to optimally incorporate the gradient information of all constraints simultaneously.

**Algorithm 2:**
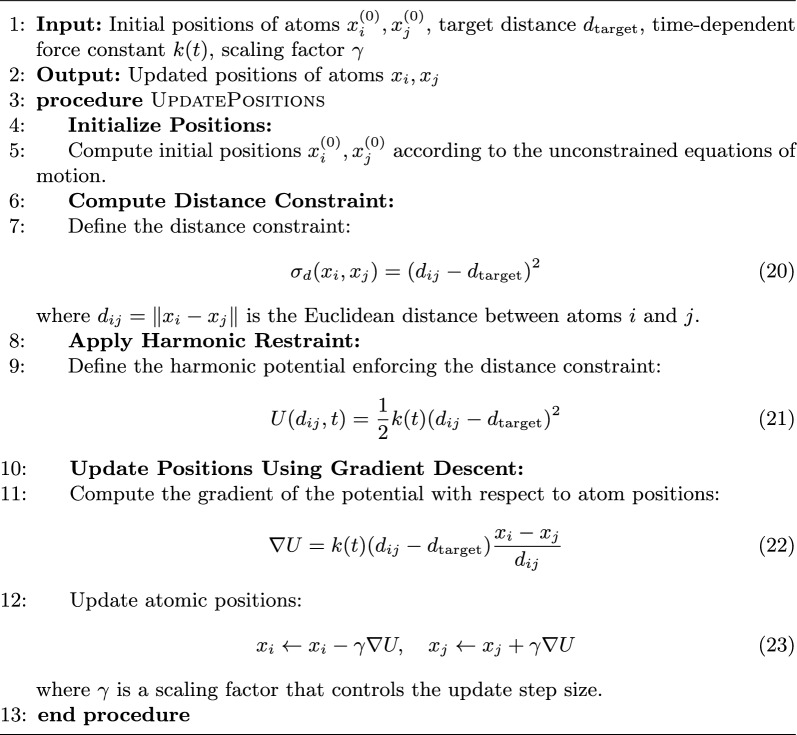
Incorporating Harmonic Restraint Potentials with Distance Constraints

### Time depenent constraint ODE algorithm

We incorporate constraints into the diffusion process by dynamically adjusting the upper and lower bounds that define each geometric constraint. At the beginning of the generation process ($t = 0$), these bounds are set to very large values, effectively imposing no (or very weak) constraints on the system. Over time, the allowable range tightens, eventually converging to the strict constraint values at $t = T$. This ensures that early in the diffusion process, the model explores a broad configuration space, while later steps refine configurations to satisfy physically meaningful constraints.

Formally, let $\sigma (x)$ be a scalar function measuring a geometric property (e.g., a bond distance, angle, or dihedral) that must lie within a certain range:

$$\begin{aligned} \sigma _{\textrm{lower}}(t) \;\le \; \sigma (x) \;\le \; \sigma _{\textrm{upper}}(t). \end{aligned}$$

We define time-dependent bounds,

$$\begin{aligned} \sigma _{\textrm{lower}}(t) \quad \text {and}\quad \sigma _{\textrm{upper}}(t), \end{aligned}$$

which interpolate from loose constraints at $t=0$ to the final strict constraints at $t=T$. The interpolation is governed by a sigmoid (logistic) schedule, $s(t)$, of the form:

24 $$\begin{aligned} s(t) = \frac{1}{1 + e^{-\beta (t - t_0)}}, \end{aligned}$$

where $\beta$ controls the steepness (sharpness) of the transition, and $t_0$ is the midpoint of the transition. For $t \ll t_0$, $s(t)\approx 0$, indicating a nearly unconstrained regime, whereas for $t \gg t_0$, $s(t)\approx 1$, indicating fully tightened constraints.

To parameterize the time-dependent bounds, we define:

25 $$\begin{aligned} \sigma _{\textrm{lower}}(t)= & \sigma _{\textrm{lower}}^{(\infty )} + \bigl (\sigma _{\textrm{lower}}^{(0)} - \sigma _{\textrm{lower}}^{(\infty )}\bigr )\bigl [1 - s(t)\bigr ], \end{aligned}$$

26 $$\begin{aligned} \sigma _{\textrm{upper}}(t)= & \sigma _{\textrm{upper}}^{(\infty )} + \bigl (\sigma _{\textrm{upper}}^{(0)} - \sigma _{\textrm{upper}}^{(\infty )}\bigr )\bigl [1 - s(t)\bigr ]. \end{aligned}$$

Here:

- $\sigma _{\textrm{lower}}^{(0)}$ and $\sigma _{\textrm{upper}}^{(0)}$ are the initial bounds, often set to $-\infty$ and $+\infty$, respectively.
- $\sigma _{\textrm{lower}}^{(\infty )}$ and $\sigma _{\textrm{upper}}^{(\infty )}$ are the final strict constraint bounds.
- $s(t)$ transitions smoothly from $0$ (loose bounds) to $1$ (tight bounds).

As $t$ progresses through the diffusion steps, the bounds converge to a narrow range around the desired physical value (e.g., a target bond distance). When these upper and lower bounds coincide, the constraint effectively becomes holonomic (strict equality). If a finite interval is maintained, the constraint is non-holonomic, allowing fluctuations within that range.

This scheduling approach facilitates that early diffusion steps are unconstrained (or weakly constrained), promoting broad exploration in the molecular configuration space. As the bounds tighten, the system’s distribution gradually collapses onto physically valid configurations, guided by the geometric constraints. The sigmoid function $(24)$ ensures a continuous, differentiable, and bijective transition.

This effectively creates a coupled system of differential equations similar to those seen in [30].

Although, the diffusion model introduces noise which breaks invertiblilty, one can always use the same score and solve with a black box ODE solver [23] to obtain a Neural ODE.

### Geometric diffusion for molecular conformer generation

To evaluate the effectiveness of our method, we employ GeoDiff, a molecular conformer generation technique based on diffusion processes. GeoDiff [31] is a state-of-the-art method for generating molecular conformations that utilizes a denoising diffusion probabilistic model. It transforms simple Gaussian distributions into the distribution of possible molecular structures.

#### Modular implementation

One of the significant advantages of our approach is that it can be implemented in a purely modular fashion, requiring no additional training. The original pretrained model can be used without modification, as the constraints are enforced through the noise projection and adjustment steps. This modular approach allows for flexibility in applying different sets of constraints to various molecules without altering the underlying model. Users can specify different initial and final constraint values and schedules depending on the requirements of the molecular system being studied.

## Deriving consistent constraints from molecular conformers

In order to study the performance of both Neural SHAKE and Guidance methods, we need to generate a set of consistent constraints in which the generative process is constrained by. Consider a molecule with *N* atoms. We denote its configuration by

$$\begin{aligned} x \;\in \; {\mathbb {R}}^{3N}, \end{aligned}$$

where *x* encodes the Cartesian coordinates of all atoms. Our objective is to enforce a set of constraints

$$\begin{aligned} \{\sigma _a(x) = 0\}_{a=1}^A \quad \text {or}\quad \{\sigma _{\textrm{lower},a}(x) \le \sigma _a(x) \le \sigma _{\textrm{upper},a}(x)\}_{a=1}^A, \end{aligned}$$

that capture essential geometric features such as distances, angles, or dihedrals. In the simplest case, each $\sigma _a(x)$ is a scalar function of *x* (e.g., the difference between an interatomic distance and its target value). We say a configuration *x* satisfies the constraints if:

$$\begin{aligned} \sigma _a(x) = 0\quad (\text {strict}), \quad \text {or} \quad \sigma _{\textrm{lower},a}(x) \le \sigma _a(x) \le \sigma _{\textrm{upper},a}(x)\quad (\text {bounded}), \end{aligned}$$

*for all* $$a\in \{1,\dots ,A\}$$.

If the constraints are linear (e.g., fixed covalent bond lengths), imposing additional constraints does not typically invalidate existing ones, and solutions remain straightforward to obtain. However, many molecular constraints—such as angles, torsion preferences, or non-bonded interactions—are nonlinear. In this setting, each new constraint can potentially negate the feasible region defined by the others; thus, picking constraints that do not co-occur in real molecular geometries may yield a system of equations with no solutions.

We define the *feasible set* of configurations under constraints $\{\sigma _a\}_{a=1}^A$ as:

$$\begin{aligned} {\mathcal {F}} \;=\; \bigl \{\, x \in {\mathbb {R}}^{3N} \;\bigl |\; \sigma _a(x) = 0\;\text {(or bounds satisfied)},\;\forall \, a \bigr \}. \end{aligned}$$

A key concern is whether ${\mathcal {F}}$ is *non-empty*: if the constraints are mutually incompatible, ${\mathcal {F}} = \emptyset$. Even if ${\mathcal {F}} \ne \emptyset$, the resulting configurations may be high-energy (i.e., geometrically feasible yet thermodynamically unlikely) unless the constraints correspond to physically realistic molecular features.

A collection of constraints is called *consistent* if:

- *Feasibility* $${\mathcal {F}} \ne \emptyset$$.
- *Physical Validity* Points $x \in {\mathcal {F}}$ exhibit *low-energy* (or at least physically reasonable) molecular conformations.

Given the nonlinear interdependence of geometric terms, constraints that appear plausible *in isolation* may jointly produce *no real solutions* or correspond to energetically unfavorable conformations. Therefore, to handle multiple molecular systems without handcrafting each constraint set, we seek an **automated** mechanism to generate *consistent* constraints.

### Generating consistent constraints

*1. Sampling Conformers.* We first gather a set of molecular conformers $\{x^{(m)}\}_{m=1}^M$ using:

- *In silico* toolkits such as RDKit, which sample physically plausible conformers.
- A generative diffusion model (or another probabilistic model) trained to output low-energy molecular structures.

Each $x^{(m)}$ is a point in ${\mathbb {R}}^{3N}$ that *should* lie in or near a local minimum of the molecular potential energy surface.

*2. Geometric Feature Extraction.* For each conformer, we measure geometric features:

$$\begin{aligned} \sigma _a\bigl (x^{(m)}\bigr ), \end{aligned}$$

such as *interatomic distances*, *valence angles*, or *torsion angles*. We record each measurement across all *M* conformers.

*3. Statistical Analysis & Clustering.* We analyze the distributions of these features across the sampled set:

$$\begin{aligned} \bigl \{\sigma _a(x^{(1)}),\, \sigma _a(x^{(2)}),\,\dots ,\,\sigma _a(x^{(M)})\bigr \}. \end{aligned}$$

We then cluster or partition these values to identify “modes” or tightly grouped sets of geometries. For each mode, we extract *characteristic bounds* or target values:

$$\begin{aligned} \sigma _{\textrm{lower},a} \;\le \; \sigma _a(x) \;\le \; \sigma _{\textrm{upper},a}, \end{aligned}$$

that appear frequently in actual conformations. By defining constraints in terms of ranges or strict values that *co-occur* within real conformers, we ensure a higher probability that all constraints can be simultaneously satisfied.

*4. Selecting Consistent Constraints.* From the clustered data, we choose subsets of constraints that consistently appear in the same cluster(s). For example, if a certain set of distance and angle constraints commonly co-occur in multiple conformers, they likely constitute a consistent set. By contrast, combining constraints from widely separated clusters might yield an infeasible or energetically unrealistic system.

To provide an intuitive understanding of the methodology, we include two visualizations: 1) an analysis of the constraints derived from clustering and 2) a PCA-based visualization with density information of the conformer space. In these visualizations we use a 15 chain carbon molecule and generate 500 conformations with rdkit. For the rest of the analysis, the conformations are generated with Geodiff.

### Methods of constraint set generation

Here we describe how to use clustering methods to generate consistent constraints, an alternative Principal Component Analysis method can be seen in the Appendix D.

Given a molecule with *n* atoms, let ${\textbf{D}}_c$ represent the pairwise distance matrix of the *c*-th conformer, where $c = 1, \dots , C$ and *C* is the total number of generated conformers from any desired sampling method, like Geodiff. The entries of ${\textbf{D}}_c$ are denoted as $d_c(i, j)$, representing the distance between atoms *i* and *j* in the *c*-th conformer. To analyze the conformers, we flatten the upper triangular elements of each distance matrix ${\textbf{D}}_c$ into a feature vector ${\textbf{x}}_c \in {\mathbb {R}}^m$, where $m = \frac{n(n-1)}{2}$ is the total number of unique atomic pairs.

The set of feature vectors $\{{\textbf{x}}_c\}_{c=1}^C$ is then used as input for clustering. Specifically, we apply *k*-means clustering to partition the conformers into *N* clusters, where *N* represents the number of local minima in the conformational space. Let ${\mathcal {C}}_k$ denote the set of conformers in the *k*-th cluster. For each cluster, we compute the mean distance vector:

$$\begin{aligned} \bar{{\textbf{x}}}_k = \frac{1}{|{\mathcal {C}}_k|} \sum _{c \in {\mathcal {C}}_k} {\textbf{x}}_c, \end{aligned}$$

and compare it to the global mean distance vector:

$$\begin{aligned} \bar{{\textbf{x}}} = \frac{1}{C} \sum _{c=1}^C {\textbf{x}}_c. \end{aligned}$$

The significance of each atomic pair (*i*, *j*) within a cluster is quantified by the absolute difference between the cluster mean and the global mean:

$$\begin{aligned} \varDelta _k(i, j) = |{\bar{x}}_k(i, j) - {\bar{x}}(i, j)|. \end{aligned}$$

We rank all atomic pairs within each cluster by $\varDelta _k(i, j)$ and select the top *T* pairs with the highest significance. These pairs, along with their corresponding mean distances ${\bar{x}}_k(i, j)$, form the set of constraints for the *k*-th cluster.

The process can be understood as identifying structural features that differentiate conformers within each local minimum of the molecule’s conformational space. By clustering conformers, we capture groups of similar geometries, which correspond to local minima. Within each cluster, the mean distances $\bar{{\textbf{x}}}_k$ represent the characteristic geometry of that local minimum.

Comparing the cluster mean distances to the global mean distances highlights the atomic pairs that vary the most between clusters. These pairs are likely to correspond to flexible or distinctive regions of the molecule, making them excellent candidates for constraints.

By focusing on the most significant atomic pairs within each cluster, this approach generates constraints that capture the key structural differences between local minima, ensuring that the constraints are the k most informative constraints that describe a cluster. As well, they reflect the inherent variability of the molecule, as they are derived from conformational sampling.

These constraints can be used in further conformer generation or molecular optimization tasks to ensure that sampled structures remain consistent with the observed conformational space, enabling more reliable exploration of the molecule’s potential energy landscape.

**Fig. 3:**
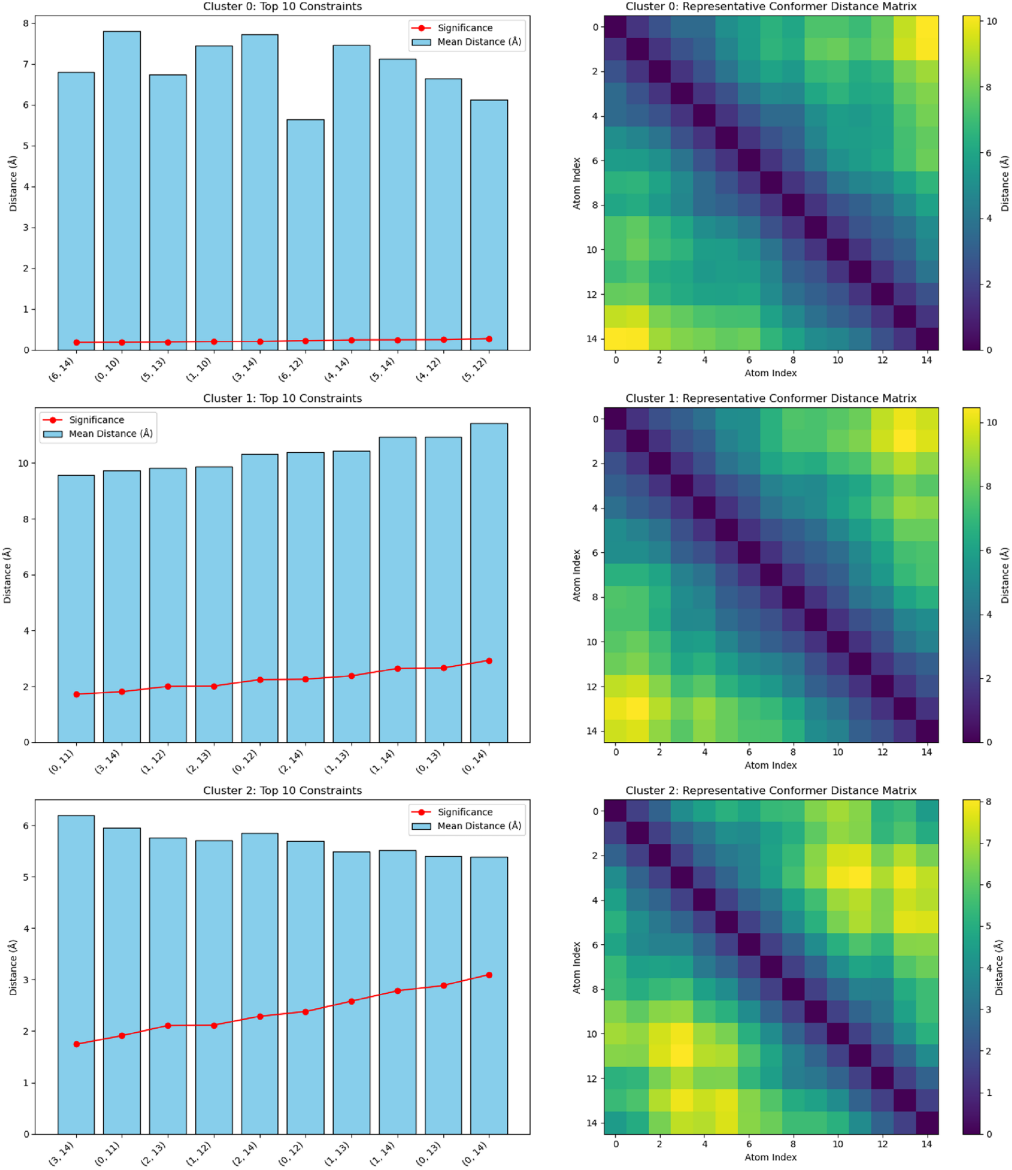
Bar plots and heatmaps showing the most significant atom pair constraints and the representative conformer, of undecane, distance matrix for each identified cluster. This visualization highlights the top constraints that are characteristic of local minima in the conformational space

The left panel in Figure 3 presents bar plots of the most significant atom pair constraints for each cluster, ranked by their deviation from global mean distances. These constraints are derived from the clustering of conformer feature vectors, capturing the characteristic structural features of each local minimum. The right panel shows heatmaps of the distance matrices for representative conformers of the corresponding clusters.

**Fig. 4:**
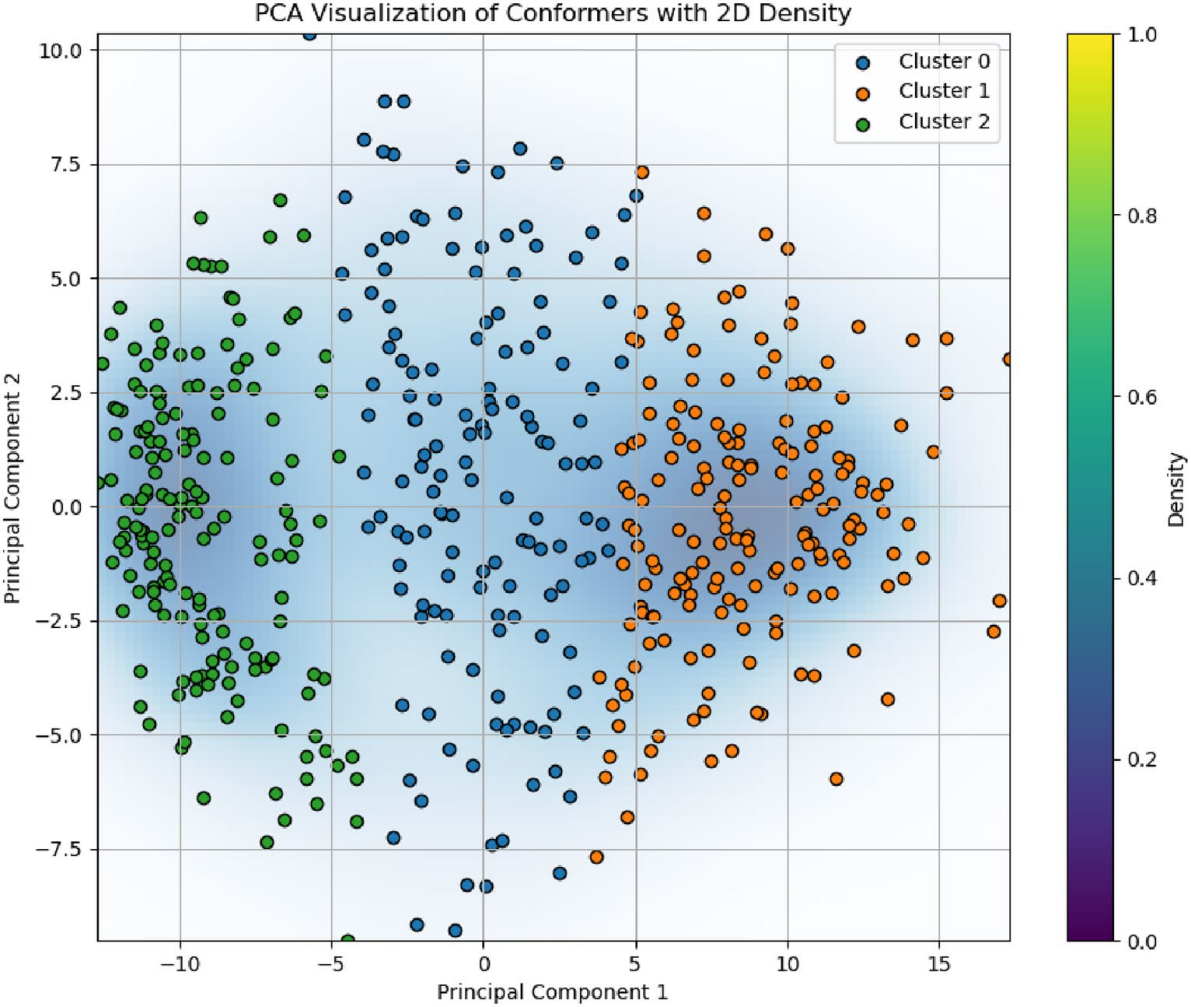
PCA-based visualization of the conformer feature vectors with a 2D density plot. This plot shows the distribution of conformers across two principal components, with clusters highlighted and density contours providing additional insight into the data distribution

Figure 4 illustrates the distribution of conformers of undecane across two principal components, highlighting the clusters identified during analysis. The background density contours provide insight into regions of higher conformer density, which correspond to well-sampled areas of the conformational space. These figures allow us to see that the constraints are derived from clusters of conformers that represent distinct regions of the geometric spaces. The yellow areas in the heatmaps shows where the interatomic distances are larger. We can see that among the clusters there is a variance in the profile of these distances. For instance, the bottom-most cluster represents a molecule that folds back on itself while the others are more extended. We can then look at which constraints are the most important for these conformer clusters.

**Fig. 5:**
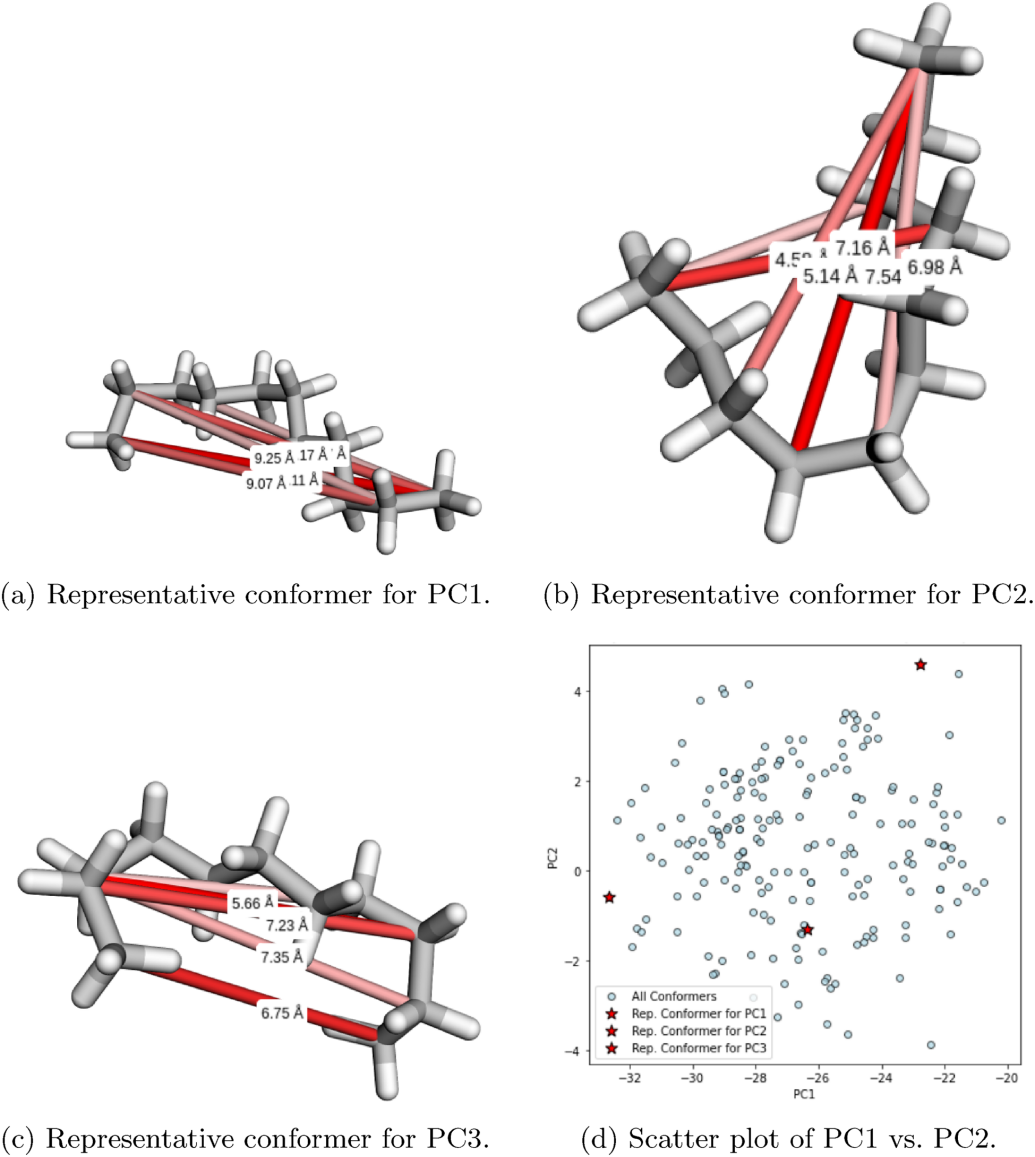
Visualization of the top three principal components derived from pairwise distance-based PCA, Appendix D. In each of (**a**), (**b**), and (**c**), we show the representative conformer chosen for that principal component, highlighting only the first few most significant distances. Each distance is drawn as a cylinder, with darker red corresponding to higher PCA loading (i.e., greater contribution to overall variance). In (**d**), all conformers are projected into PC1–PC2 space, with the red stars marking the representative conformers

Figure 5 illustrates how principal component analysis (PCA) identifies key structural variations in the conformational ensemble. The first three panels (a, b, and c) show representative conformers chosen for the top three principal components (PC1, PC2, and PC3). Each structure highlights the most significant interatomic distances, where darker red cylinders indicate greater significance in contributing to variance along the given principal component. Only the top-ranked distances are visualized to maintain clarity.

**Fig. 6:**
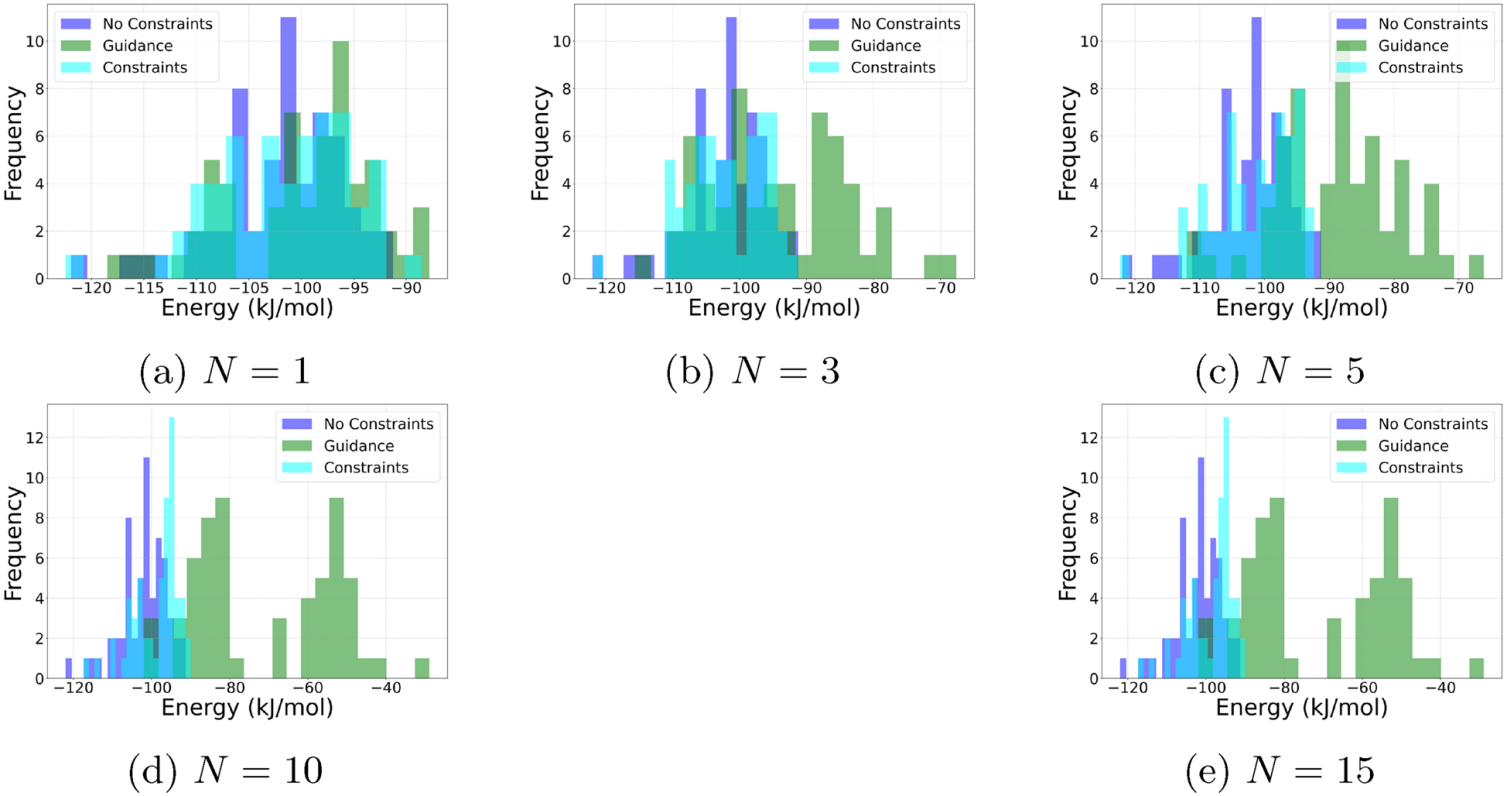
Visualization of the energy distribution of NC(N)=N under different conditions. The figure illustrates the results for no constraints, guidance, and Neural Shake (Constraints) with *N* number of constraints, where *N* corresponds to the values 1, 3, 5, 10, and 15

## Performance analysis of neural shake vs. guidance

In this section, we analyze the effectiveness of Neural Shake in comparison to the Guidance method using a series of quantitative evaluations.

To evaluate the effectiveness of our method, we employ GeoDiff, a molecular conformer generation technique based on diffusion processes. GeoDiff [31] is a state-of-the-art method for generating molecular conformations that utilizes a denoising diffusion probabilistic model. It transforms simple Gaussian distributions into the distribution of possible molecular structures.

As shown in Figure 6, we present the energy distribution of the simple molecule NC(N)=N under different generative conditions. The unconditional sampling, represented as *no constraints*, utilizes the GeoDiff method without additional guidance. Two methods, Neural Shake and Guidance, are then employed to incorporate *N* constraints into the generative process. The results indicate a general trend where, as the number of constraints *N* increases, Neural Shake consistently outperforms the guidance method in optimizing energy distribution. As *N* increases, Guidance gradually makes more and more higher energy structures while attempting to remain in the constrained feasible region. However, Neural SHAKE remains much closer to the unperturbed energies. This highlights the efficacy of Neural Shake in accommodating constraints within the generative process compared to traditional guidance approaches.

**Fig. 7:**
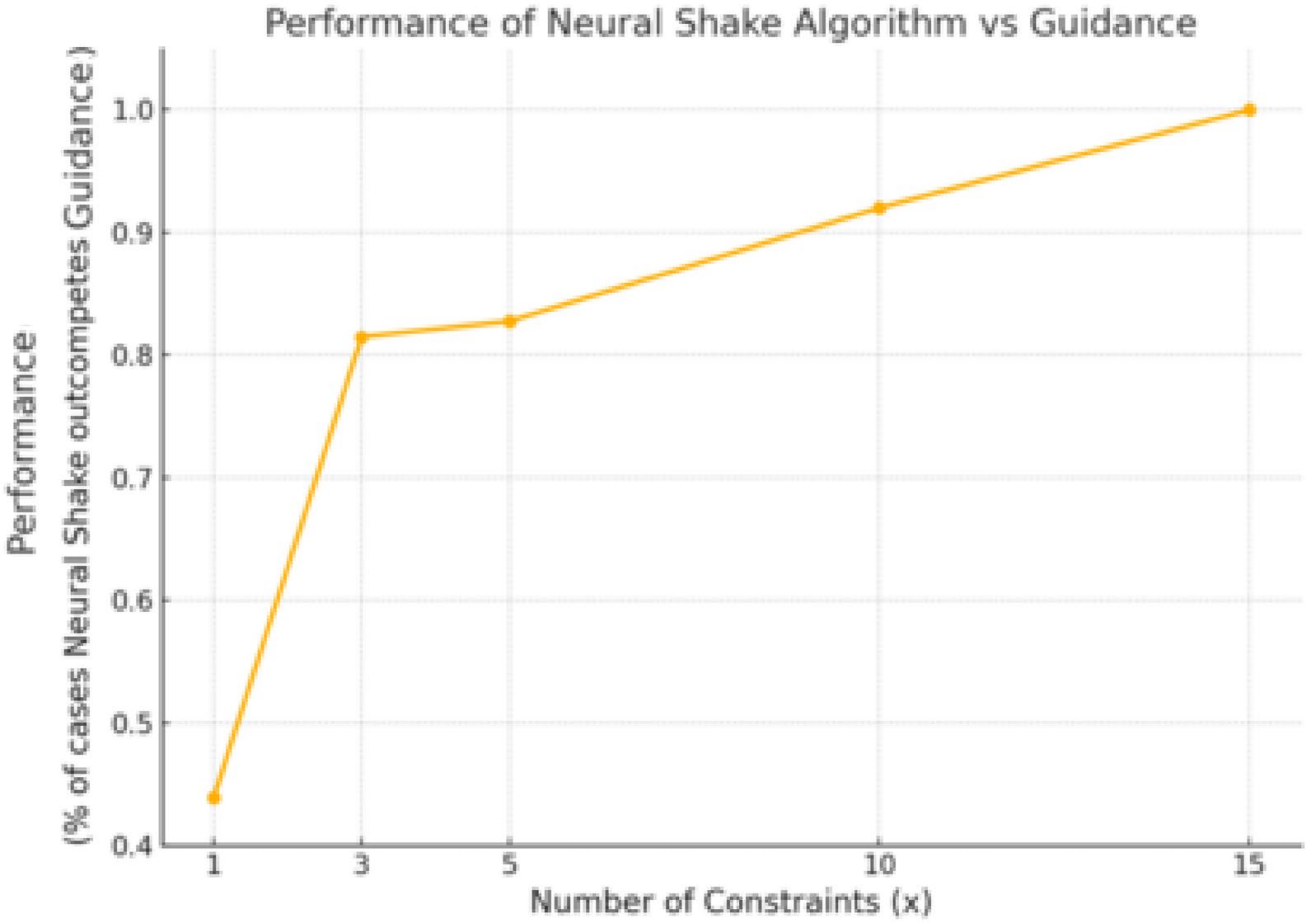
Performance of Neural Shake Algorithm compared to guidance as the number of constraints *N* increases. The performance metric represents the percentage of cases where Neural Shake outcompetes guidance. While the numbers suggest guidance performs well, they often overestimate its performance because guidance frequently fails to satisfy the desired constraints, which implicitly improves the energies of the resulting molecules using Guidance

**Fig. 8:**
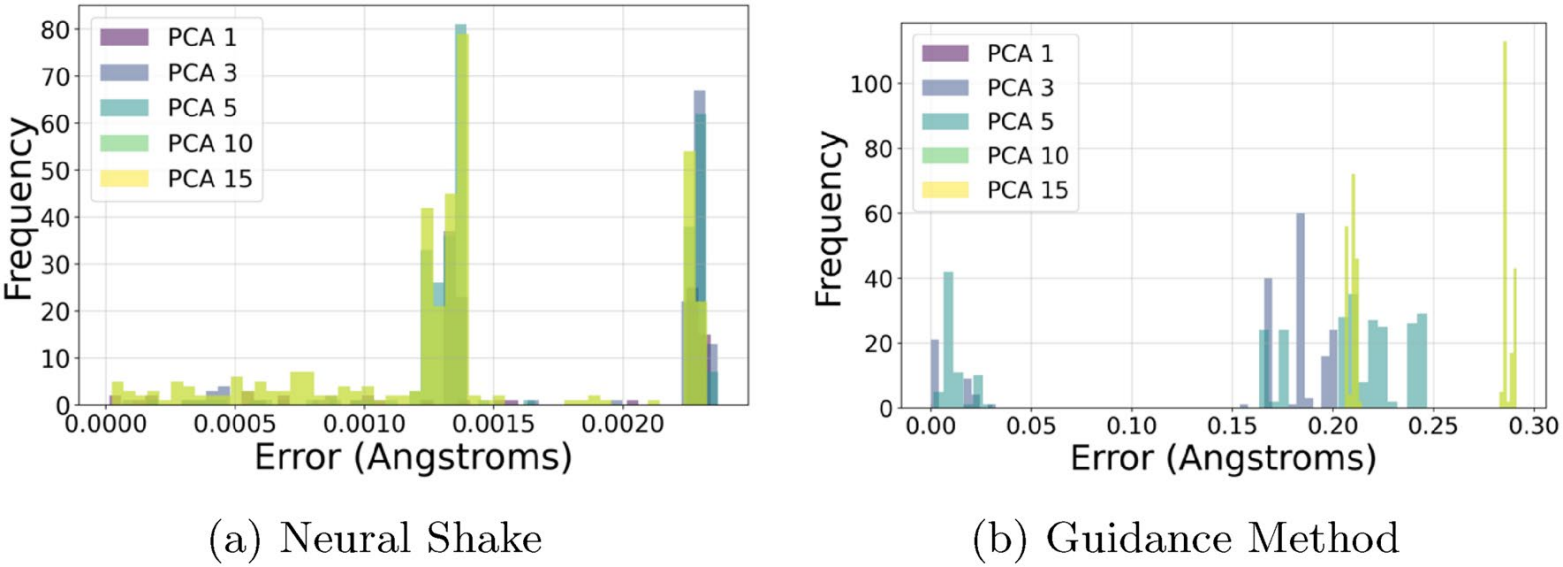
Comparison of Neural Shake and Guidance Method for constraint satisfaction. Image (**a**) demonstrates significantly fewer errors in constraint satisfaction compared to image (**b**) both sampling the conformations of molecule NC(N)=N . The Neural Shake algorithm employs a hyperparameter for constraint violation acceptance, which is set to 0.003 throughout. This tight control over violations enables Neural Shake to outperform the Guidance method in consistently adhering to the constraints

**Fig. 9:**
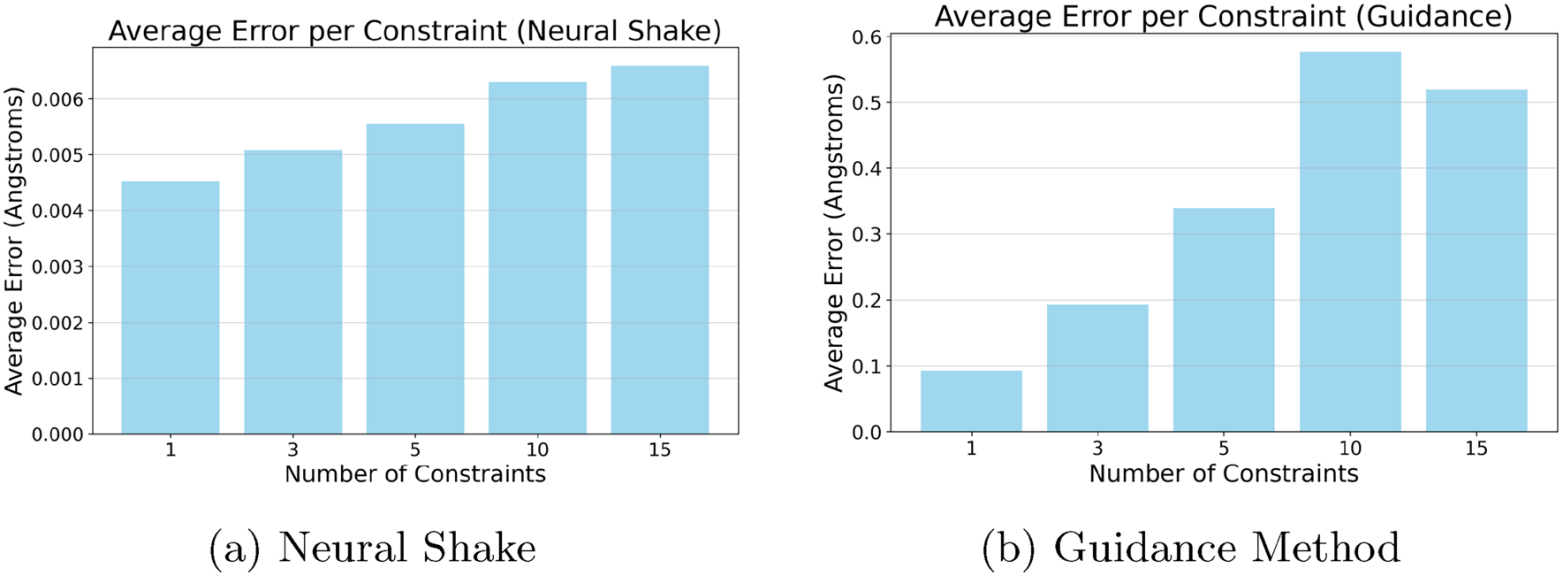
Comparison of average error per number of constraints between Neural Shake and Guidance methods. Image (**a**) shows the performance of Neural Shake, which achieves nearly 100 times better average error per constraint compared to the Guidance method shown in image (**b**). This demonstrates the superior ability of Neural Shake to maintain low error rates as the number of constraints increases

Figure 7 evaluates the overall performance of the Neural Shake Algorithm in comparison to Guidance as the number of constraints, $N$, increases. The assessment is conducted on a diverse set of 80 molecules randomly selected from DrugBank [32], ensuring a balanced range of molecular flexibility and size. The figure presents the percentage of cases where Neural Shake achieves lower energy distributions than Guidance by comparing the average energies across generated batches. While the numerical results may suggest that Guidance performs competitively for small N, this interpretation is misleading. Guidance frequently fails to satisfy constraints accurately, meaning that its energy evaluations do not necessarily correspond to correctly constrained molecular structures. In contrast, Neural Shake not only improves adherence to constraints but also achieves more physically meaningful energy distributions, highlighting its reliability.

Figure 8 provides a direct visual comparison of constraint satisfaction between Neural Shake and Guidance. Subfigure (a) illustrates that Neural Shake achieves significantly fewer constraint violations, leading to well-formed molecular structures. Conversely, in subfigure (b), Guidance exhibits more frequent constraint failures, resulting in inconsistent molecular geometries. This discrepancy arises because Neural Shake explicitly incorporates all constraints into an optimized global update, while Guidance applies independent iterative updates for each constraint. Although additional iterations of Guidance may reduce constraint violations, they introduce progressively larger deviations from the intended diffusion process. These perturbations degrade energy distributions and compromise molecular stability, making Guidance less effective for generating reliable structures.

**Fig. 10:**
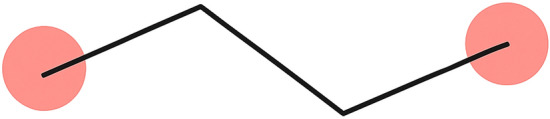
Visualization of butane with red highlights indicating the atoms partaking in the constraints

Finally, Figure 9 quantifies the constraint satisfaction accuracy by measuring the average error per constraint across both methods. Neural Shake maintains significantly lower error rates, achieving up to 100 times greater precision in constraint adherence compared to Guidance. This highlights its robustness, particularly as the number of constraints increases. The ability of Neural Shake to maintain high accuracy while preserving stable energy distributions makes it particularly well-suited for molecular generation tasks where precise geometric control is essential Fig. 10.

Across all evaluations, Neural Shake consistently surpasses Guidance in key performance metrics: constraint satisfaction, energy minimization, and stability within the diffusion process. Unlike Guidance, which struggles with constraint enforcement at scale, Neural Shake remains robust and efficient, making it a more reliable approach for generating physically accurate molecular conformations under constraints.

## Results: geodiff generating butane

**Fig. 11:**
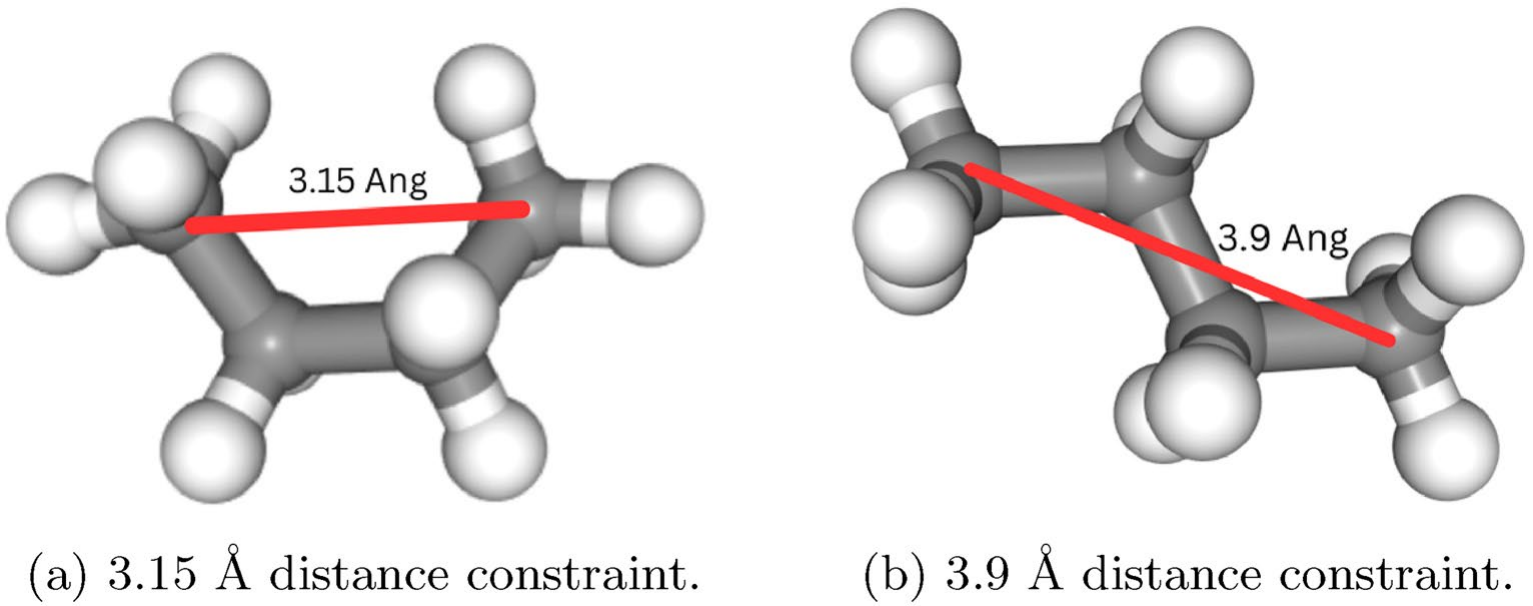
Applying 3.9 vs 3.15 Å distance constraints between the first and fourth carbon atoms generates the staggered vs eclipsed conformation of butane

**Fig. 12:**
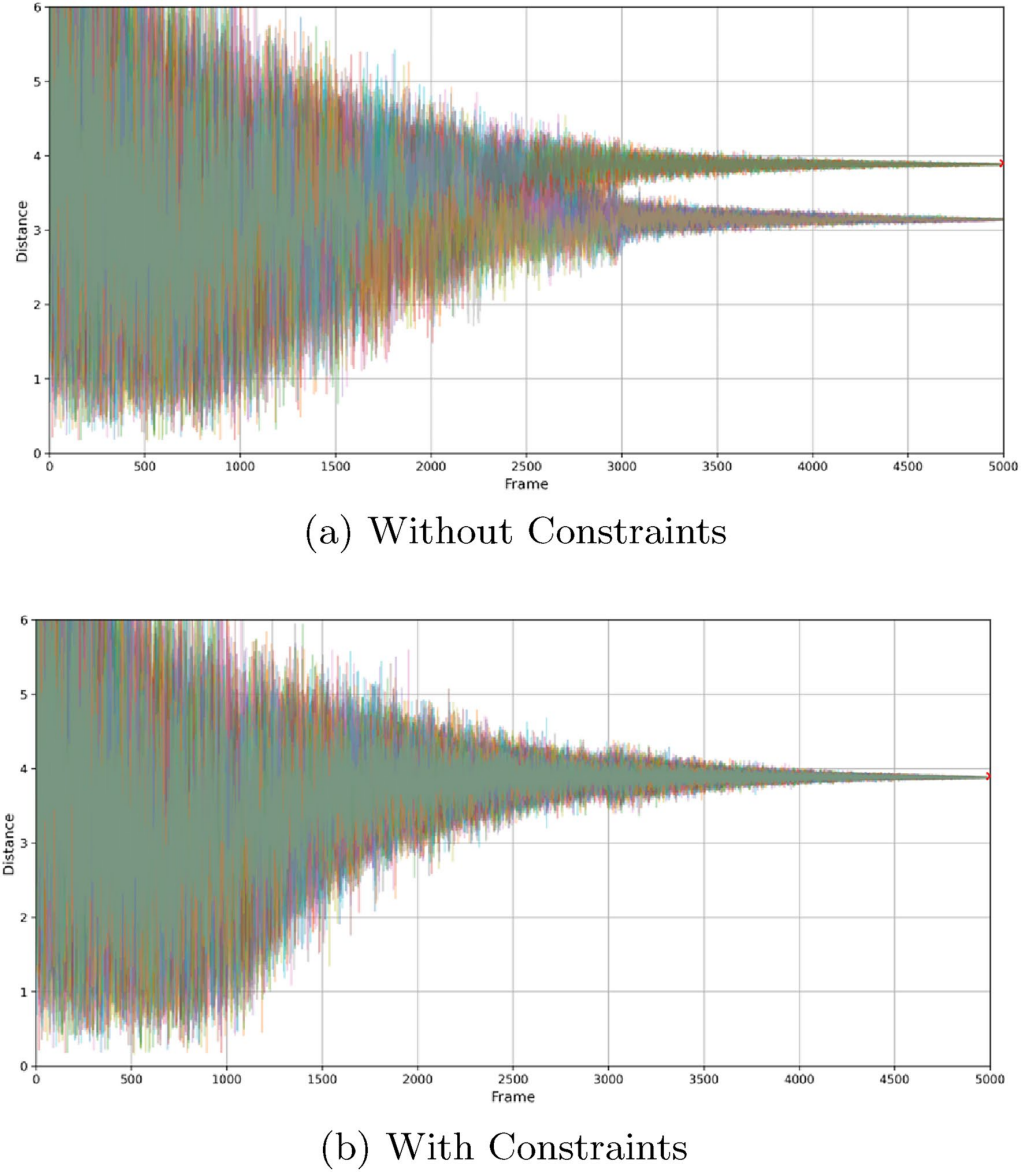
Distance between the first and fourth carbon atoms over time in butane generation for all batch elements.** a** Without constraints showing two distances indicating staggered and eclipsed conformations.** b** With constraints showing convergence to a single distance indicating guided generation to the most likely conformation

**Fig. 13:**
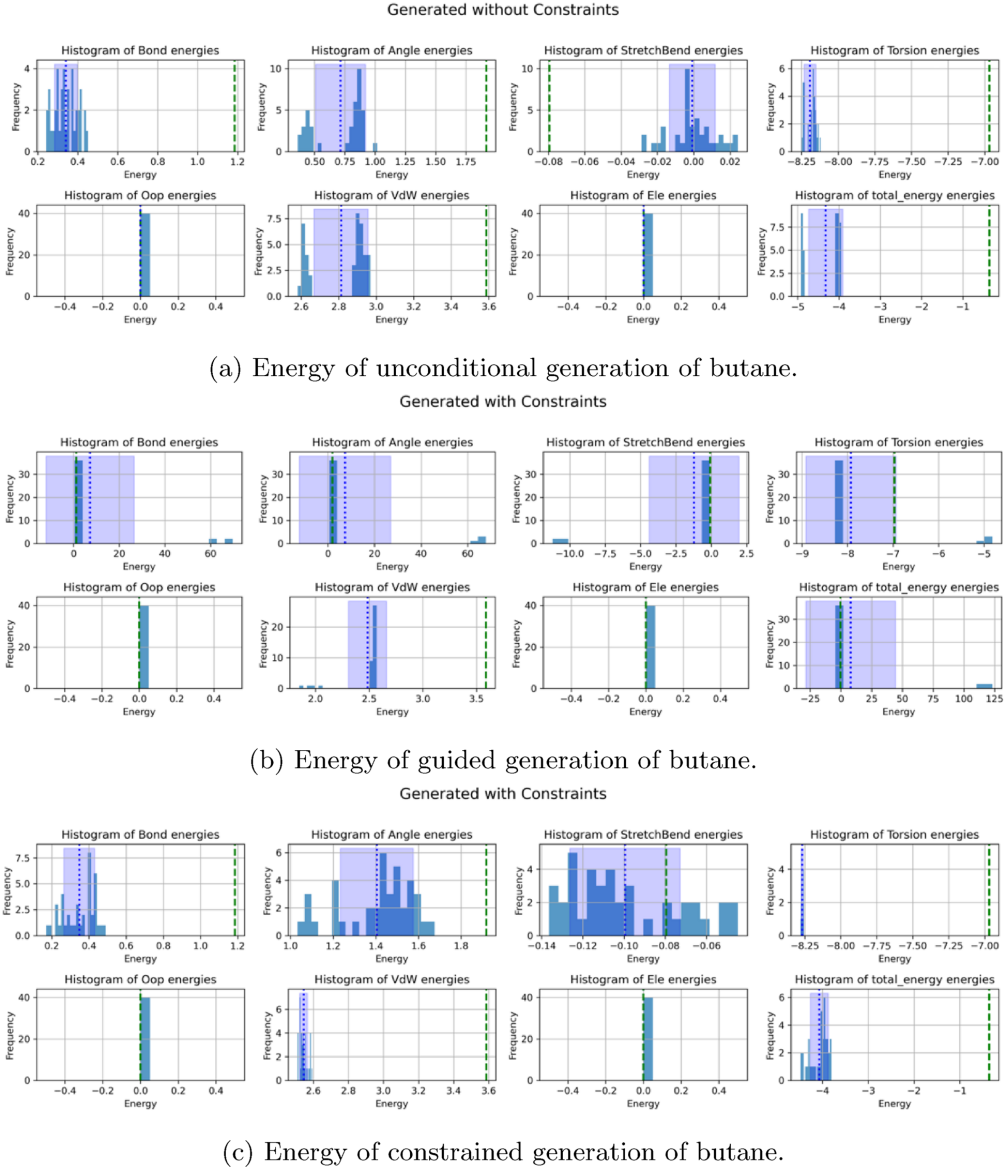
Comparison of energies in butane generation under different conditions: **a** Unconditional generation,** b** Guided generation, and** c** Constrained generation. Constraint is between the first and fourth carbon atoms with a 3.9 Anstrom distance value. We see that with constraint generation, we accurately capture the eclipsed distribution even sampling the correct Vander Waals energy distribution

To illustrate our method further, we use a simple example of butane with four carbon atoms with explicit hydrogen. Butane serves as a simple yet effective test case for evaluating the impact of constraints on molecular generation. As a four-carbon alkane, butane exhibits well-defined conformational states–primarily staggered and eclipsed–determined by the dihedral rotation of its carbon-carbon bonds. By introducing constraints during the generation process, we can selectively guide the model towards one of the two specific modes of butane’s conformational space, allowing for controlled exploration of molecular geometries. This makes butane an ideal system for assessing how constraints influence structural distributions and energy landscapes in molecular generation tasks.

By analyzing these distributions, which can be obtained for simple molecules like butane with RDKit [33], we note that the single torsion angle of the carbons of butane takes on values near 180 degrees for the staggered conformation and around 90 or 270 degrees for the eclipsed conformations. In addition, we note that these angles correspond almost uniquely to a distance between the first and fourth carbon atom of 3.9 and 3.15 Å, respectively (Figure 11). However, a single distance constraint is not always sufficient as it may perturb the diffusion model out of distribution and generate physical artifacts like hydrogen atoms separated, from the carbon it is covalently bound to, further than the corresponding equilibrium bond distance. To mitigate this, we also include covalent bond constraints set to equilibrium bond distances of the carbon-carbon bonds obtained from RDKit.

Figure 12 illustrates the evolution of the distance between the first and fourth carbon atoms over time during butane generation under constrained and unconstrained conditions. In the unconstrained case (Figure 12a), two distinct distance distributions emerge, corresponding to the staggered and eclipsed conformations of butane. This demonstrates that the generative model is capable of correctly sampling the natural flexibility of the molecule, freely exploring both conformational states without imposed restrictions.

In contrast, the constrained case (Figure 12b) shows a clear convergence to a single distance over time. This indicates that the application of constraints effectively restricts the model to a specific conformational subpopulation, reducing variability and ensuring adherence to the imposed structural requirements. The gradual stabilization of distances highlights how constraints guide molecular generation towards controlled structural outcomes, enforcing a predetermined molecular geometry while maintaining consistency across the generated batch.

### Energy comparisons of butane generation

We now examine the energy distributions of butane generated using three different methods: unconditional generation, guidance-based generation, and Neural Shake-based constrained generation. Figure 13 highlights key differences between these approaches, particularly in their ability to differentiate conformational modes while maintaining physically meaningful structures.

Unconditional generation accurately samples the expected staggered and eclipsed conformations without introducing unphysical artifacts. The energy distribution remains well-behaved, reflecting the natural flexibility of butane without any imposed constraints. This confirms that the generative model, when left unconstrained, can correctly reproduce the inherent conformational landscape of butane.

Guidance-based generation introduces a bias toward one conformation but occasionally produces incorrect, high-energy structures. While it generally succeeds in distinguishing the two conformational modes, its independent constraint updates sometimes lead to physically unrealistic configurations, with a subset of samples exceeding 100 kcal/mol. This suggests that guidance, while effective in shifting the distribution, lacks robustness in ensuring global physical consistency across constraints.

Neural Shake, by contrast, exhibits the highest level of constraint enforcement, generating only the single conformation specified by the imposed constraints. Unlike guidance, it does so without introducing high-energy artifacts, ensuring that all generated structures remain physically realistic. This demonstrates the effectiveness of Neural Shake in strictly guiding molecular generation toward constrained subpopulations while maintaining structural integrity.

These results illustrate the relative advantages and limitations of each method. While both guidance and Neural Shake enable constraint-driven generation, only Neural Shake enforces constraints without compromising physical plausibility. This makes it a more reliable tool for targeted molecular generation, particularly in scenarios where strict adherence to structural constraints is necessary.

## Constrained generation of larger molecules

**Fig. 14:**
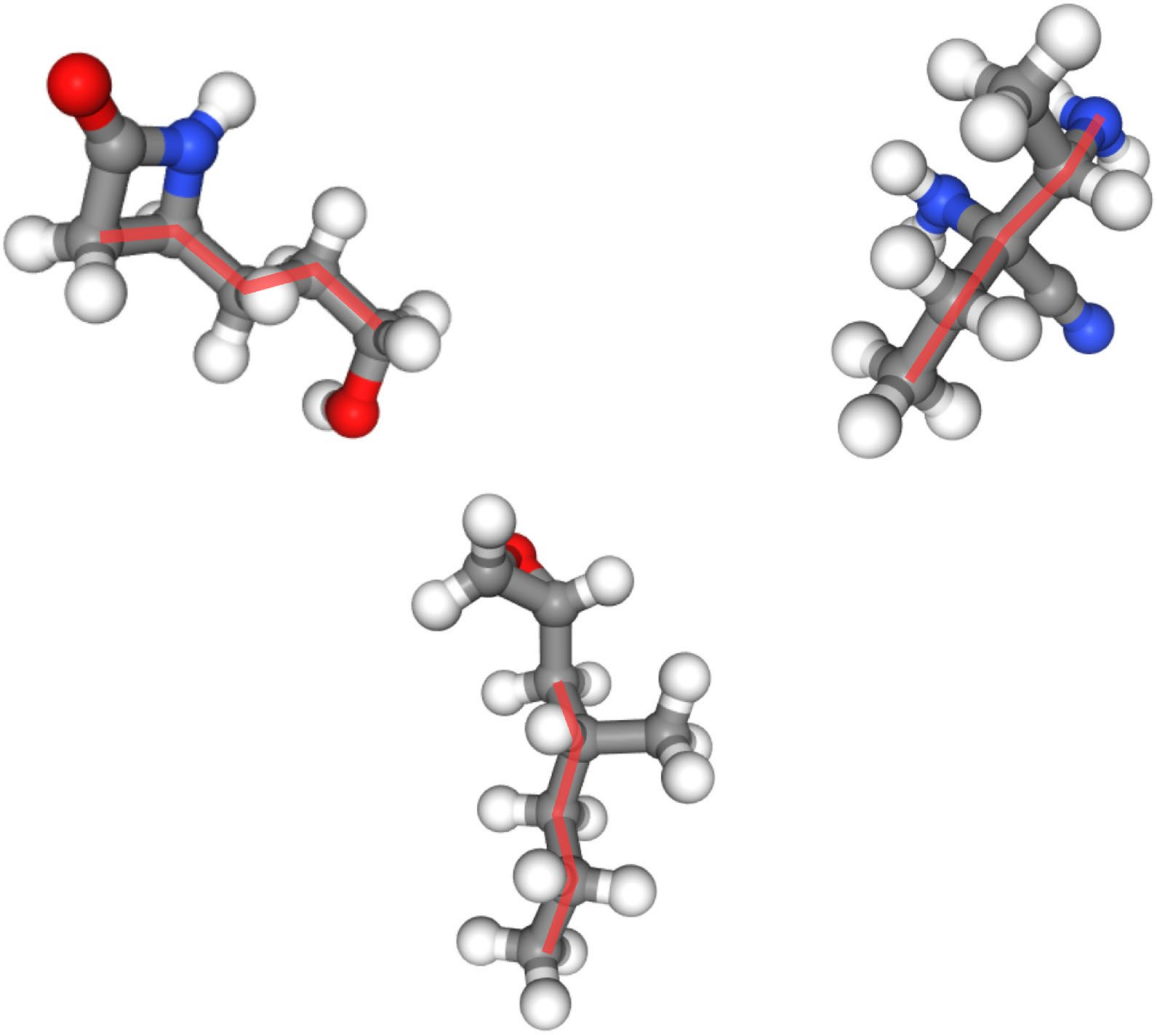
Pairwise constraints amidst 5 carbon atoms in staggered positions, where constraints are obtained from reference data examples. In all cases, constraints are satisfied with no artifacts

While previous sections focused on the simple case of butane, we now examine the effectiveness of our method in larger molecular systems. Figure 14 illustrates the application of constraints to molecules beyond butane, demonstrating that our approach successfully enforces sub-conformations even in more complex structures.

In these examples, the imposed constraints ensure that a five-carbon fragment adopts a staggered conformation, as highlighted by the red outlines. This constraint enforcement is non-trivial, as larger molecules introduce additional flexibility, steric interactions, and competing energetic factors that can potentially disrupt local structural preferences. Despite these complexities, our method consistently maintains the desired sub-conformation, indicating its robustness in preserving specific molecular geometries across different systems.

## Analysis of neural SHAKE and guidance perturbation of diffusion processes

In the previous section, we suggest that Neural SHAKE outperforms Guidance methods because the former is able to dynamically adjust the correction to a magnitude that minimally satisfies the constraint sets. In contrast, Guidance methods work by adding to the diffusion process an extra potential that cannot easily be manipulated to predefined values like constraints can. This can be illustrated with an example. If the diffusion process is pushing two atoms apart greater than the potential is pushing them together, then the two atoms will never be brought together. However, with Neural SHAKE, the forces are dynamically modified, and thus increased, in order to satisfy the constraint. In this section, we give results about the magnitude of perturbations throughout the diffusion process with Neural SHAKE and Guidance methods. We see that the former is mostly active during the middle stages of the diffusion process while the constraints need to continually correct the noise added to the diffusion process. While Guidance can be seen to have a more extended influence. We show this by comparing the perturbations introduced by Neural SHAKE and Guidance methods while generating butane conformations.

### Pairwise distance constraints

Let $x \in {\mathbb {R}}^{3N}$ denote the Cartesian coordinates of *N* atoms. A typical pairwise distance constraint between atoms *i* and *j* can be written as

$$\begin{aligned} \sigma _{ij}(x) \;=\; \Vert x_i - x_j \Vert \;-\; d_{ij}^* \;=\; 0, \end{aligned}$$

where $d_{ij}^*$ is the target distance for that atomic pair. Denote

$$\begin{aligned} r_{ij} \;=\; \Vert x_i - x_j \Vert , \quad \nabla \sigma _{ij}(x_i) \;=\; \frac{x_i - x_j}{r_{ij}}, \quad \nabla \sigma _{ij}(x_j) \;=\; \frac{x_j - x_i}{r_{ij}}, \end{aligned}$$

assuming $$r_{ij} \ne 0$$.

### Neural SHAKE

The Neural SHAKE algorithm enforces constraints by iteratively solving for a set of Lagrange multipliers $\{\lambda _{ij}\}$ that correct any violations of $\sigma _{ij}(x) = 0$. In each iteration *n*, the update to $x_i^{(n)}$ has the form

27 $$\begin{aligned} x_i^{(n)} \;=\; x_i^{(n-1)} \;-\; \sum _{(i, j)\in {\mathcal {P}}} \lambda _{ij}^{(n-1)} \, \nabla \sigma _{ij}\bigl (x_i^{(n-1)}\bigr ), \end{aligned}$$

where ${\mathcal {P}}$ is the set of all constrained pairs. The multipliers $\lambda _{ij}^{(n-1)}$ are determined by solving a linear system that ensures each $\sigma _{ij}\bigl (x^{(n)}\bigr ) \approx 0$.

*Perturbation Magnitude.* Define the net constraint-induced correction at iteration *n* for atom *i* as

$$\begin{aligned} \varDelta x_i^{(\textrm{constraints})} \;=\; -\sum _{(i, j)\in {\mathcal {P}}} \lambda _{ij}^{(n-1)}\, \nabla \sigma _{ij}\bigl (x_i^{(n-1)}\bigr ). \end{aligned}$$

A straightforward measure of its size is the Euclidean norm

$$\begin{aligned} \bigl \Vert \varDelta x_i^{(\textrm{constraints})}\bigr \Vert \;=\; \left\| -\sum _{(i, j)\in {\mathcal {P}}} \lambda _{ij}^{(n-1)}\, \nabla \sigma _{ij}\bigl (x_i^{(n-1)}\bigr ) \right\| . \end{aligned}$$

Large corrections indicate that the atom’s coordinates deviate substantially from the prior (unconstrained) motion, potentially overshadowing the diffusion model’s learned score if used in a generative pipeline.

### Guidance

In the Guidance case, we embed these distance constraints into a harmonic potential:

$$\begin{aligned} U_{\text {dist}}(x) \;=\; \sum _{(i, j)\in {\mathcal {P}}} \frac{k_{ij}}{2} \bigl (\Vert x_i - x_j\Vert - d_{ij}^*\bigr )^2, \end{aligned}$$

where $k_{ij} > 0$ are force constants. A gradient-based update with step size $\gamma$ for atom *i* takes the form

28 $$\begin{aligned} x_i \;\leftarrow \; x_i \;-\; \gamma \, \nabla _i U_{\text {dist}}(x), \quad \nabla _i U_{\text {dist}}(x) \;=\; \sum _{(i, j)\in {\mathcal {P}}} k_{ij} \bigl (r_{ij} - d_{ij}^*\bigr ) \, \frac{x_i - x_j}{r_{ij}}. \end{aligned}$$

In analogy with Neural SHAKE, we can define

$$\begin{aligned} \varDelta x_i^{(\textrm{harmonic})} \;=\; -\gamma \, \nabla _i U_{\text {dist}}(x). \end{aligned}$$

Its norm,

$$\begin{aligned} \bigl \Vert \varDelta x_i^{(\textrm{harmonic})}\bigr \Vert \;=\; \left\| -\gamma \sum _{(i, j)\in {\mathcal {P}}} k_{ij} \bigl (r_{ij} - d_{ij}^*\bigr ) \frac{x_i - x_j}{r_{ij}} \right\| , \end{aligned}$$

indicates how strongly the penalty-based method shifts the system toward the target distances at each update.

**Fig. 15:**
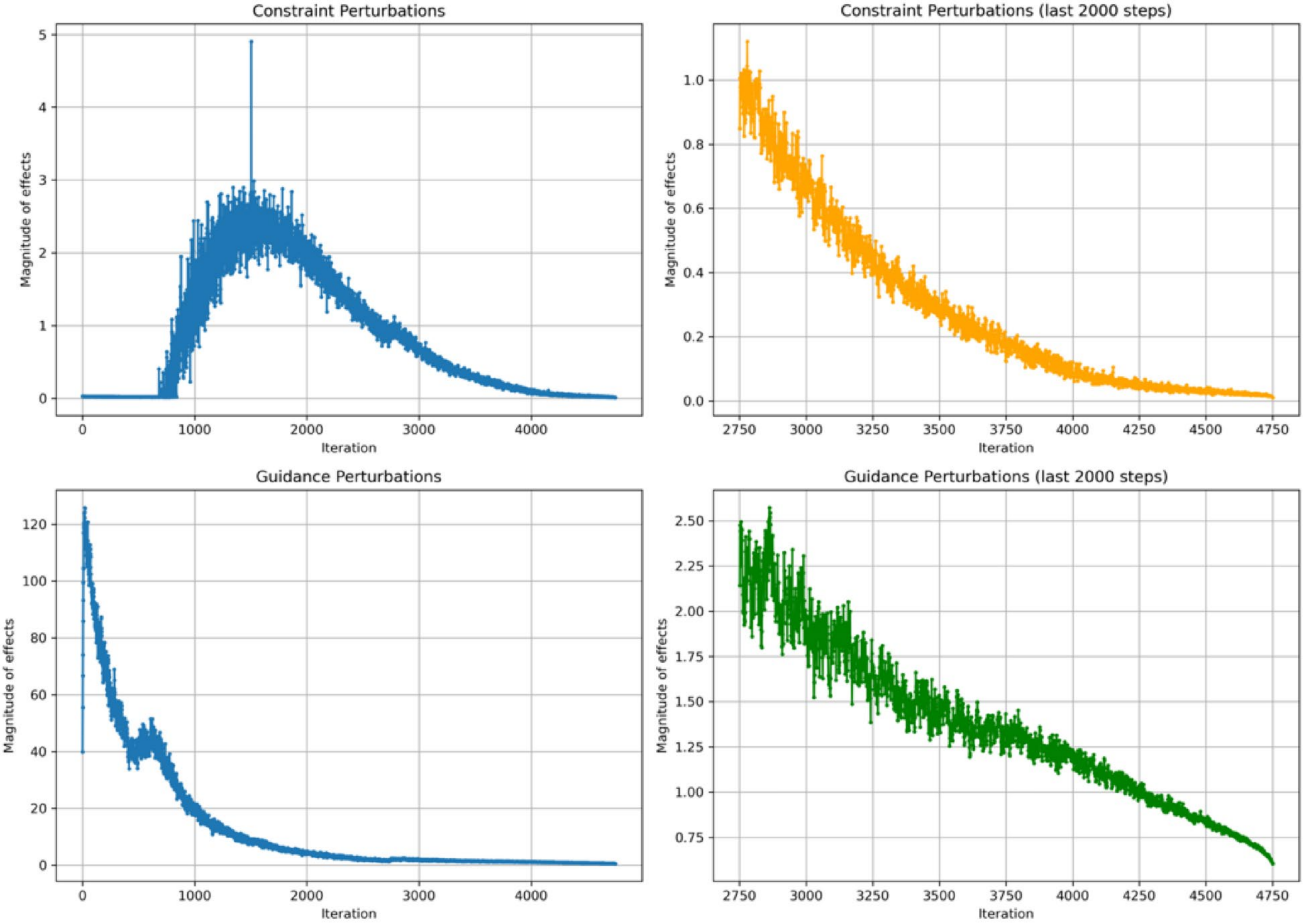
Magnitude of effects over 5000 iterations for Neural Shake and Guidance of Butane. Top left: Magnitude of Constraint Perturbations over 5000 iterations, showing a relatively low and decreasing trend. Top right: Zoomed-in view of the last 2000 iterations for Constraint Perturbations, highlighting the reduction in perturbation effects. Bottom left: Magnitude of Guidance Perturbations over 5000 iterations, exhibiting significantly higher magnitudes, particularly at the beginning. Bottom right: Zoomed-in view of the last 2000 iterations for Guidance Perturbations, providing a clearer view of the diminishing perturbations over time

Figure 15 compares the magnitude of perturbations introduced by Neural SHAKE and Guidance during the generation of butane conformers. As shown, constraint-induced perturbations lead to smaller deviations in dynamics than harmonic potentials, particularly at later stages of the diffusion process. The perturbations from Neural SHAKE are more localized and diminish once the system approaches the constraint-defined subspace. In contrast, the perturbations from harmonic potentials are more consistent and large in magnitude, especially at the beginning of the simulation. This difference in perturbation sizes highlights that constraints, when properly enforced, lead to more stable and controlled updates, while the larger perturbations of harmonic potentials can introduce greater fluctuations in generated molecular structures.

### Computational costs comparison

The Neural SHAKE algorithm enforces constraints iteratively at each time step. Let $N$ denote the total number of atoms and $N_c$ the number of constraints applied to the system. The computational steps involve computing gradients, which scales as $O(N_c \cdot N)$, solving for Lagrange multipliers, which requires $O(N_c^3)$, and updating atomic positions, which contributes another $O(N_c \cdot N)$ per iteration. This results in an overall cost per time step of

$$\begin{aligned} O(I \cdot (N_c \cdot N + N_c^3)), \end{aligned}$$

where $I$ represents the number of iterations needed for constraint satisfaction.

The Guidance method, which enforces constraints through harmonic potentials rather than explicit Lagrange multipliers, has a lower computational complexity. The force calculations scale as $O(N_c \cdot N)$, and the integration of equations of motion contributes an additional $O(N)$, leading to a total cost per time step of

$$\begin{aligned} O(N_c \cdot N + N). \end{aligned}$$

Since Neural SHAKE explicitly solves a system of constraints, its cost scales with $O(N_c^3)$ due to the Lagrange multiplier step. However, as long as the number of constraints $N_c$ remains moderate, the computational burden is manageable. Additionally, because constraint satisfaction typically involves only local updates rather than global operations over the entire system, the method remains feasible in practical applications.

## Conclusion

In this work, we introduced Neural SHAKE, a novel approach for integrating geometric constraints into neural differential equation frameworks using diffusion-based generative models. By projecting updates onto constrained subspaces, our method enables the generation of molecular conformations that strictly adhere to physical constraints, offering a significant improvement over conventional probabilistic guidance techniques. Through a rigorous mathematical formulation, we demonstrated that Neural SHAKE effectively enforces non-linear holonomic constraints while maintaining minimal perturbations to the generative process.

Our experiments confirm that Neural SHAKE outperforms existing guidance methods in key areas like higher accuracy in constraint satisfaction, maintaining lower-energy molecular conformations more consistently, reducing perturbation magnitudes during diffusion steps, ensuring greater stability. By bridging classical constraint enforcement methods with modern generative modeling, Neural SHAKE provides a scalable and modular solution that can be readily incorporated into existing diffusion models without additional retraining. Future work may look into more general types of geometric expressions to constrain such as probabilistic, or molecular property based constraints.

## Data Availability

The data and documentation supporting this study are provided as supplementary information, including the analysis data of the molecules. Additionally, the constraint algorithm is made available in a Python file along with the consistent constraint generation pipeline.

## Additional methodology descriptions

*Number of diffusion steps.* Throughout the manuscript we use the symbol *m* to denote the *total number of discrete reverse-diffusion steps* (i.e. time intervals) employed by the generative process. Unless stated otherwise, *all* experiments are run with the standard setting $m = 5000$, mirroring the default schedule used in GeoDiff models.

*RDKit* Where RDKit’s ETKDG algorithm was employed to generate initial conformers: we requested up to 50 trial conformations per molecule using the default distance geometry parameters [Riniker2015]. Each RDKit conformer was then energy-minimized using the MMFF94 force field (500 iterations or until convergence).

*Symmetry preservation.* Neural SHAKE’s constraint-projection step *preserves the global* $\textrm{SE}(3)$ *symmetry*. The underlying score network is $\textrm{SE}(3)$-equivariant by construction, relying only on rotation-/translation-invariant features such as interatomic distances and angles. Moreover, the holonomic constraints we enforce are themselves invariant to rigid motions. Consequently, if a global rotation $R \in \textrm{SO}(3)$ or translation $t \in {\mathbb {R}}^{3}$ is applied to an intermediate conformation ${\textbf{x}}$, the projected configuration satisfies

$$\begin{aligned} P(R{\textbf{x}} + t)\;=\; R\,P({\textbf{x}}) + t , \end{aligned}$$

where $P(\cdot )$ denotes the SHAKE projection onto the constraint manifold. In other words, the entire Neural SHAKE pipeline operates consistently on the quotient space ${\mathbb {R}}^{3N}/\textrm{SE}(3)$ and does *not* break the rotational or translational equivariance guaranteed by the score model.

### Well-posedness of the projection step

Projecting an unconstrained configuration ${\textbf{x}}\in {\mathbb {R}}^{3N}$ onto the nearest point of the constraint manifold ${\mathcal {M}}$ is locally well defined only when ${\textbf{x}}$ lies inside the *normal injectivity radius* of ${\mathcal {M}}$ and the associated constraint Jacobian has full rank. Outside this neighbourhood–i.e. in the vicinity of the *cut locus*, where the nearest constrained point is not unique–the SHAKE projection may converge to a sub-optimal solution or fail to converge altogether.

Typical degeneracies include (i) three atoms becoming colinear when a bond–angle constraint enforces non-colinearity, and (ii) four atoms approaching coplanarity so that a dihedral angle is ill-conditioned. In both situations the Jacobian of the constraint equations becomes nearly singular, hampering Newton–style updates.

In our implementation we observed no projection failures because *(a)* diffusion trajectories are initialised from equilibrium structures that already satisfy all constraints, and *(b)* each reverse-diffusion step adds only a small noise increment, keeping the correction $\varDelta {\textbf{x}}$ within the linear regime where the Jacobian is well conditioned.

## PCA and clustering methods

After generating 100 conformers for each molecule, we first aligned all conformers to a common reference frame (using a least-squares fit on the heavy atoms) to remove arbitrary rotations/translations. We then performed PCA on the aligned coordinates across the ensemble. We have added a statement that a scree plot was examined to determine how many principal components to consider: in our tests, the first 3 components typically accounted for about 80–90 percent of the variance. Thus, 2D PCA plots provide a reasonable visualization of the major conformational degrees of freedom. We note that PCA is a linear projection; a nonlinear dimensionality reduction like Multidimensional Scaling (MDS) based on RMSD distances could capture fine-grained structural differences. We chose PCA for simplicity and because it sufficed to separate distinct clusters in our data. By clustering the generated conformers, we identify groups of structures that correspond to the same broad potential energy basin. We call each such cluster a “unique conformational state” or local minimum (since within each cluster, conformers relax to a similar geometry upon energy minimization). Our evaluation counts how many known low-energy basins were discovered by each method.

## Equivalence of distance constraints to all other geometric constraints

In molecular systems and 3D point clouds, spatial configurations are often expressed using geometric constraints such as bond lengths, bond angles, and dihedral angles. Here, we reillustrate that any geometric property of a system of points can be rewritten solely in terms of pairwise distances.

### Fundamental representation of a point cloud

Consider a set of $N$ points ${\textbf{r}}_i \in {\mathbb {R}}^3$, whose structure is fully described by the Euclidean distances:

$$\begin{aligned} d_{ij} = \Vert {\textbf{r}}_i - {\textbf{r}}_j\Vert . \end{aligned}$$

The distance matrix $D = [d_{ij}]$ uniquely determines the geometric configuration up to rigid transformations.

### Geometric constraints in terms of distances

#### Bond lengths

A bond length constraint is already given directly by the distance $d_{ij}$.

#### Bond angles

For three atoms $(i,j,k)$, the bond angle $\theta _{ijk}$ satisfies the law of cosines:

$$\begin{aligned} \cos \theta _{ijk} = \frac{d_{ij}^2 + d_{jk}^2 - d_{ik}^2}{2 d_{ij} d_{jk}}. \end{aligned}$$

Thus, bond angles are expressible purely in terms of distances.

#### Dihedral angles

For four atoms $(i,j,k,l)$, the dihedral angle $\phi _{ijkl}$ is given by a determinant formula involving distances:

$$\begin{aligned} \cos \phi _{ijkl} = \frac{ \det {\textbf{G}} }{\sqrt{\det \mathbf {G_1} \det \mathbf {G_2}}} \end{aligned}$$

where ${\textbf{G}}$ is a Gram matrix of vectors formed from the distances $d_{ij}, d_{jk}, d_{kl}, d_{ik}, d_{jl}, d_{il}$.

### Theoretical justification

#### Theorem 2

(Fundamental Theorem of Distance Geometry) A set of $N$ points in ${\mathbb {R}}^3$ is uniquely determined (up to rigid transformations) by its pairwise distances, provided the distance matrix satisfies Euclidean embedding conditions.

#### Proof

See [34, 35] for details. $\square$

## Alternative PCA-based constraint selection

An alternative way to identify structurally significant interatomic distances is through a principal component analysis (PCA) of the pairwise distance features extracted from a large ensemble of conformers. Like clustering-based methods, PCA seeks to uncover a small subset of critical distances that best characterize the conformational variability. However, rather than highlighting differences among local minima, PCA focuses on directions of maximal variance across *all* sampled structures.

Given a molecule with *n* atoms, let ${\textbf{D}}_c \in {\mathbb {R}}^{n \times n}$ denote the pairwise distance matrix for the *c*-th conformer, where $c = 1, \dots , C$. Each matrix entry $d_c(i, j)$ represents the distance between atoms *i* and *j*. To transform each conformer into a feature vector, we flatten only the upper-triangular elements of ${\textbf{D}}_c$ (since ${\textbf{D}}_c$ is symmetric) into a vector ${\textbf{x}}_c \in {\mathbb {R}}^m$, with

$$\begin{aligned} m = \frac{n(n-1)}{2}. \end{aligned}$$

This procedure yields a set of *C* feature vectors:

$$\begin{aligned} \{{\textbf{x}}_c\}_{c=1}^C,\quad {\textbf{x}}_c \in {\mathbb {R}}^m, \end{aligned}$$

where each ${\textbf{x}}_c$ encodes all unique pairwise distances for conformer *c*.

To capture how these distance features co-vary, we construct the sample covariance matrix:

$$\begin{aligned} \mathbf {\varSigma } \;=\; \frac{1}{C-1} \sum _{c=1}^C \bigl ({\textbf{x}}_c - \bar{{\textbf{x}}}\bigr )\bigl ({\textbf{x}}_c - \bar{{\textbf{x}}}\bigr )^{\textsf{T}}, \end{aligned}$$

where

$$\begin{aligned} \bar{{\textbf{x}}} \;=\; \frac{1}{C} \sum _{c=1}^C {\textbf{x}}_c \end{aligned}$$

is the global mean. We then perform an eigenvalue decomposition,

$$\begin{aligned} \mathbf {\varSigma }\,{\textbf{v}}_\ell \;=\; \lambda _\ell \,{\textbf{v}}_\ell , \quad \ell = 1, \dots , m, \end{aligned}$$

and sort the eigenvalues $\{\lambda _\ell \}$ in descending order so that the largest ones correspond to the principal directions of maximum variance. The associated eigenvectors $\{{\textbf{v}}_\ell \}$ are the principal components, each of which is a vector in ${\mathbb {R}}^m$ indicating how strongly each pairwise distance contributes to the variance in that direction.

Let ${\textbf{v}}_\ell$ denote the $\ell$-th principal component. Its *i*-th entry, $v_\ell (i)$, quantifies how strongly the *i*-th distance feature influences that component. We define the *significance* of the pair (*i*, *j*) under component $\ell$ as

$$\begin{aligned} s_\ell (i, j) \;=\; \bigl |v_\ell (\text {index}(i,j))\bigr |, \end{aligned}$$

where $\text {index}(i,j)$ maps the (*i*, *j*) pair to the corresponding coordinate in the flattened vector. A larger $s_\ell (i,j)$ indicates that (*i*, *j*) contributes more strongly to variation along ${\textbf{v}}_\ell$. Consequently, we can identify the top *T* interatomic distances,

$$\begin{aligned} \text {TopPairs}(\ell ) \;=\; \underset{(i,j)}{\mathrm {arg\,top\,}T}\, s_\ell (i, j), \end{aligned}$$

which best capture the conformational diversity along the $\ell$-th principal component.

Distances that appear in the top *T* for multiple principal components are typically those that co-vary the most across the ensemble. Chemically, they often correspond to bond lengths or angles that drive major structural rearrangements. Because PCA draws on all sampled conformers, it inherently focuses on global variability, and the selected distances can guide conformer generation, structure refinement, or validation tasks by ensuring that these key degrees of freedom are adequately explored.

To anchor these selected distances to actual geometries, let ${\textbf{x}}_c \in {\mathbb {R}}^m$ be the flattened distance vector for the *c*-th conformer, and let ${\textbf{v}}_\ell \in {\mathbb {R}}^m$ denote the $\ell$-th principal component. We define the *projection* of conformer *c* onto component $\ell$ as

$$\begin{aligned} p_{c,\ell } \;=\; {\textbf{x}}_c^{\textsf{T}}\,{\textbf{v}}_\ell . \end{aligned}$$

Among all conformers, we then select a representative one, $c^*$, whose projection $p_{c^*,\ell }$ is particularly large in magnitude (or otherwise near a desired percentile). By reading off the corresponding pairwise distances directly from the chosen conformer’s 3D geometry, we obtain physically realizable constraints that reflect the principal mode of variation. In contrast, constraints taken from raw averages $\bar{{\textbf{x}}}$ may not correspond to any single valid conformer and can inadvertently violate geometric consistency. Consequently, this PCA-based selection method offers a complementary perspective to clustering-based approaches, highlighting global modes of conformational variance rather than discrete differences between local minima.

### Principal component analysis results

Figure 5 provides an overview of how principal component analysis (PCA) captures the conformational diversity of the sampled molecule. The first three panels (a, b, c) depict representative conformers for each of the top three principal components, highlighting only the first few most significant interatomic distances. Darker shades of red indicate higher significance in driving conformational variation, whereas lighter shades correspond to less significant distances. In each image, the labeled cylinders represent these selected distances. Figure 5 (d) shows a two-dimensional scatter plot of all sampled conformers in the space of PC1 and PC2, illustrating how the data points cluster. The red stars in that plot indicate the representative conformers selected for each principal component.

## Measure-theoretic view: constrained probability density

A key insight is to describe how *imposing constraints* transforms the *probability measure* of *X*(*t*). Let ${\widetilde{p}}(x,t)$ be the density of the **unconstrained** SDE. When we restrict *X*(*t*) to satisfy ${\varvec{\sigma }}(x,t)={\varvec{0}}$, the resulting distribution *collapses* onto ${\mathcal {M}}(t)$ and is no longer a full-dimensional density in ${\mathbb {R}}^d$. Instead, it becomes supported on a $(d-A)$-dimensional manifold.

### Dirac deltas and the gram determinant

The distribution can be formally written as

29 $$\begin{aligned} p_{\textrm{constrained}}(x,t) \;=\; {\widetilde{p}}(x,t)\; \delta \!\bigl ({\varvec{\sigma }}(x,t)\bigr ) \; \sqrt{\det \!\Bigl ( \nabla _x {\varvec{\sigma }}(x,t)\,\bigl [\nabla _x {\varvec{\sigma }}(x,t)\bigr ]^\top \Bigr )} \;\Big /\;Z(t), \end{aligned}$$

where

$$\begin{aligned} \delta \!\bigl ({\varvec{\sigma }}(x,t)\bigr ) \;=\; \prod _{a=1}^A \delta \!\bigl (\sigma _a(x,t)\bigr ) \end{aligned}$$

is a product of Dirac deltas enforcing the constraints, and

$$\begin{aligned} \sqrt{\det \!\Bigl (\nabla _x {\varvec{\sigma }}(x,t)\,\bigl [\nabla _x {\varvec{\sigma }}(x,t)\bigr ]^\top \Bigr )} \end{aligned}$$

is the *Jacobian factor* (Gram determinant). The denominator

$$\begin{aligned} Z(t) \;=\; \int _{{\mathbb {R}}^d} {\widetilde{p}}({\tilde{x}},t)\; \delta \bigl ({\varvec{\sigma }}({\tilde{x}},t)\bigr )\; \sqrt{\det \!\Bigl (\nabla _x {\varvec{\sigma }}({\tilde{x}},t)\,\bigl [\nabla _x {\varvec{\sigma }}({\tilde{x}},t)\bigr ]^\top \Bigr )} \;d{\tilde{x}} \end{aligned}$$

ensures normalization to 1.

#### Remark 1

(Surface Measure in ${\mathcal {M}}(t)$) By the *coarea formula* (Theorem 4 below), we know

$$\begin{aligned} \int _{{\mathbb {R}}^d} \varPhi (x)\; \delta \bigl ({\varvec{\sigma }}(x,t)\bigr ) \;\sqrt{\det \!\Bigl (\nabla _x {\varvec{\sigma }}(x,t)\,[\nabla _x {\varvec{\sigma }}(x,t)]^\top \Bigr )}\,dx \;=\; \int _{x\in {\mathcal {M}}(t)} \varPhi (x)\,dS_x, \end{aligned}$$

where $dS_x$ is the induced surface measure on the submanifold ${\mathcal {M}}(t)$. Thus, (29) indeed represents a valid density *restricted to* ${\mathcal {M}}(t)$.

#### Theorem 3

(Constrained Density on a Moving Manifold) Let ${\widetilde{X}}(t)\in {\mathbb {R}}^d$ solve the SDE with density ${\widetilde{p}}(x,t)$. Impose constraints ${\varvec{\sigma }}(x,t)=0$ of rank *A*. Then the **constrained** process $X(t)\in {\mathcal {M}}(t)$ has a probability distribution given for any test function $\varPhi$ by

$$\begin{aligned} {\mathbb {E}}[\varPhi (X(t))]&\;=\; \int _{{\mathcal {M}}(t)} \varPhi (x)\,\underbrace{p_{\textrm{constrained}}(x,t)}_{\text {density on }{\mathcal {M}}(t)}\,dS_x, \\ p_{\textrm{constrained}}(x,t)&\;=\; \frac{ {\widetilde{p}}(x,t)\, \delta \bigl ({\varvec{\sigma }}(x,t)\bigr )\, \sqrt{\det \!\Bigl (\nabla _x {\varvec{\sigma }}(x,t)\,\bigl [\nabla _x {\varvec{\sigma }}(x,t)\bigr ]^\top \Bigr )} }{ \displaystyle \int {\widetilde{p}}({\tilde{x}},t)\, \delta \bigl ({\varvec{\sigma }}({\tilde{x}},t)\bigr )\, \sqrt{\det \!\Bigl (\nabla _x {\varvec{\sigma }}({\tilde{x}},t)\,[\nabla _x {\varvec{\sigma }}({\tilde{x}},t)]^\top \Bigr )} \,d{\tilde{x}} }. \end{aligned}$$

That is, the density $p_{\textrm{constrained}}(\cdot ,t)$ is supported *only* on ${\mathcal {M}}(t)$ and integrates to 1 over that manifold (with respect to $dS_x$).

#### Proof

Fix any Borel set $\varOmega \subseteq {\mathcal {M}}(t)$ and consider the event $\{\,X(t) \in \varOmega \}\,$ under the constrained process *X*(*t*). By construction (via the SDAE or its equivalent small-step projection method), the process *X*(*t*) remains on the manifold ${\mathcal {M}}(t)$ and is driven by the same underlying Wiener process as the *unconstrained* SDE, except that any component of motion normal to ${\mathcal {M}}(t)$ is removed (or “projected out”) at each step.

Hence, the probability of *X*(*t*) lying in $\varOmega \subseteq {\mathcal {M}}(t)$ can be obtained by first taking the *unconstrained* density ${\widetilde{p}}(x,t)$ in ${\mathbb {R}}^d$ and then restricting attention to the set where ${\varvec{\sigma }}(x,t) = {\varvec{0}}$. Formally,

$$\begin{aligned} \textrm{Prob}\bigl [X(t)\in \varOmega \bigr ] \;=\; \int _{\varOmega \subseteq {\mathcal {M}}(t)} p_{\textrm{constrained}}(x,t)\,\underbrace{dS_x}_{\begin{array}{c} \text {surface measure on}\\ {\mathcal {M}}(t) \end{array}}. \end{aligned}$$

We aim to identify the form of $p_{\textrm{constrained}}(x,t)$ that makes this probability consistent with the procedure of “impose ${\varvec{\sigma }}(x,t)=0$ on ${\widetilde{p}}(x,t)$” in ${\mathbb {R}}^d$

- *Step 1: express the probability via delta constraints in *
  ${\mathbb {R}}^d$. Because $\varOmega \subseteq {\mathcal {M}}(t)$, enforcing ${\varvec{\sigma }}(x,t) = 0$ in the ambient space ${\mathbb {R}}^d$ can be done with the product of Dirac delta functions,
  $$\begin{aligned} \delta \bigl ({\varvec{\sigma }}(x,t)\bigr ) \;=\; \prod _{a=1}^A \delta \bigl (\sigma _a(x,t)\bigr ). \end{aligned}$$
  Thus, an *unnormalized* measure for the event $\{X(t)\in \varOmega \}$ can be written as
  $$\begin{aligned} \int _{{\mathbb {R}}^d} {\widetilde{p}}(x,t)\, {\textbf{1}}_\varOmega (x)\, \delta \bigl ({\varvec{\sigma }}(x,t)\bigr )\; \underbrace{J(x,t)}_{\text {to be determined}} \,dx, \end{aligned}$$
  where *J*(*x*, *t*) is a Jacobian factor that converts the ambient measure *dx* into a surface measure on ${\mathcal {M}}(t)$.

- *Step 2: identify the necessary Jacobian from the coarea formula* We recall the *coarea formula* in codimension *A*: for a smooth ${\varvec{\sigma }}:{\mathbb {R}}^d\rightarrow {\mathbb {R}}^A$ of full rank on $\{{\varvec{\sigma }}(x)=0\}$,
  $$\begin{aligned} \int _{{\mathbb {R}}^d} \varPhi (x)\, \delta \!\bigl ({\varvec{\sigma }}(x)\bigr )\, \sqrt{\det \!\Bigl (\nabla {\varvec{\sigma }}(x)\,\nabla {\varvec{\sigma }}(x)^\top \Bigr )} \;dx \;=\; \int _{x \in \{{\varvec{\sigma }}(x)=0\}} \varPhi (x) \;dS_x. \end{aligned}$$
  Applying this to $\varOmega \subseteq {\mathcal {M}}(t)$ at fixed *t*, we see that
  $$\begin{aligned} J(x,t) \;=\; \sqrt{\det \!\Bigl (\nabla _x {\varvec{\sigma }}(x,t)\,\bigl [\nabla _x {\varvec{\sigma }}(x,t)\bigr ]^\top \Bigr )} \end{aligned}$$
  is exactly the factor that ensures
  $$\begin{aligned} \int _{{\mathbb {R}}^d} {\widetilde{p}}(x,t)\,{\textbf{1}}_\varOmega (x)\, \delta \bigl ({\varvec{\sigma }}(x,t)\bigr )\, J(x,t) \,dx \;=\; \int _{x\in \varOmega \,\subseteq \, {\mathcal {M}}(t)} {\widetilde{p}}(x,t)\,dS_x. \end{aligned}$$
  Furthermore, one must then *normalize* this measure so that $\int _{{\mathcal {M}}(t)} p_{\textrm{constrained}}(x,t)\,dS_x = 1$.

- *Step 3: solve for* $p_{\textrm{constrained}}(x,t)$. By definition, on ${\mathcal {M}}(t)$,
  30 $$\begin{aligned} p_{\textrm{constrained}}(x,t)&= \frac{ {\widetilde{p}}(x,t)\; \delta \bigl ({\varvec{\sigma }}(x,t)\bigr )\; J(x,t) }{ \displaystyle \int _{{\mathbb {R}}^d} {\widetilde{p}}({\tilde{x}},t)\; \delta \bigl ({\varvec{\sigma }}({\tilde{x}},t)\bigr )\; J({\tilde{x}},t)\; d{\tilde{x}} } \quad \text {with} \nonumber \\ J(x,t)&= \sqrt{\det \!\Bigl (\nabla _x {\varvec{\sigma }}(x,t)\,[\nabla _x {\varvec{\sigma }}(x,t)]^\top \Bigr )}. \end{aligned}$$
  Because $\delta \bigl ({\varvec{\sigma }}(x,t)\bigr )$ is zero whenever ${\varvec{\sigma }}(x,t)\ne {\varvec{0}}$, this expression is supported only on ${\mathcal {M}}(t)$. Hence, if $\varOmega \subseteq {\mathcal {M}}(t)$, then
  $$\begin{aligned} \textrm{Prob}[X(t)\in \varOmega ] \;=\; \int _{\varOmega } p_{\textrm{constrained}}(x,t)\,dS_x, \end{aligned}$$
  matching the probability measure derived from the SDAE viewpoint (where all motion normal to ${\mathcal {M}}(t)$ is disallowed).

- *Step 4: Uniqueness* If there were a different density ${\widetilde{q}}(x,t)$ supported on ${\mathcal {M}}(t)$ that gave the same probabilities for all Borel $\varOmega \subseteq {\mathcal {M}}(t)$, it would have to coincide with $p_{\textrm{constrained}}(x,t)$ $dS_x$-almost everywhere on ${\mathcal {M}}(t)$, by definition of equality of measures. Thus $p_{\textrm{constrained}}(\cdot ,t)$ is the *unique* density restricted to ${\mathcal {M}}(t)$ that is consistent with the original unconstrained ${\widetilde{p}}(\cdot ,t)$ under the action of imposing ${\varvec{\sigma }}(\cdot ,t)=0$.

Since all steps hold for every $t\in [0,T]$, the process *X*(*t*) satisfying ${\varvec{\sigma }}(X(t),t)=0$ almost surely must have density given by the stated formula in (29). Therefore, $p_{\textrm{constrained}}(\cdot ,t)$ describes exactly how the unconstrained measure ${\widetilde{p}}(\cdot ,t)$ is restricted to the manifold ${\mathcal {M}}(t)$. $\square$

### Coarea formula in higher codimension

For completeness, we recall the coarea formula in codimension *A*. A version of it is sometimes written as:

$$\begin{aligned} \int _{{\mathbb {R}}^d} \varPhi (x)\;\delta \bigl ({\varvec{\sigma }}(x)\bigr )\,\sqrt{\det \!\bigl [\nabla {\varvec{\sigma }}(x)\,\nabla {\varvec{\sigma }}(x)^\top \bigr ]}\;dx \;=\; \int _{\{x:\,{\varvec{\sigma }}(x)=0\}} \varPhi (x)\;dS_x, \end{aligned}$$

where ${\varvec{\sigma }}:{\mathbb {R}}^d\rightarrow {\mathbb {R}}^A$ is smooth and of rank *A* on $\{{\varvec{\sigma }}(x)=0\}$, and $dS_x$ is the induced surface measure on that $(d-A)$-dimensional manifold.

#### Theorem 4

(Coarea Formula, Codimension *A*) Let ${\varvec{g}}:{\mathbb {R}}^d\rightarrow {\mathbb {R}}^A$ be sufficiently smooth with full rank *A* on the zero set $\varSigma :=\{x:{\varvec{g}}(x)=0\}$. For any $\varPhi :{\mathbb {R}}^d\rightarrow {\mathbb {R}}$ integrable,

$$\begin{aligned} \int _{{\mathbb {R}}^d} \varPhi (x)\;\prod _{a=1}^A \delta \bigl (g_a(x)\bigr ) \;\sqrt{\det \!\Bigl (\nabla {\varvec{g}}(x)\,\nabla {\varvec{g}}(x)^\top \Bigr )} \;dx \;=\; \int _{x\in \varSigma } \varPhi (x)\,dS_x, \end{aligned}$$

where $dS_x$ is the natural $(d-A)$-dimensional surface measure on $\varSigma$.

This theorem immediately justifies the form of $p_{\textrm{constrained}}$ in Theorem 3.

#### Remark 2

(Time-Dependent Version) If ${\varvec{\sigma }}(x,t)$ depends on *t*, then for each *fixed* *t*, the same coarea formula holds (treating *t* as a parameter). The manifold ${\mathcal {M}}(t)=\{x:\,{\varvec{\sigma }}(x,t)={\varvec{0}}\}$ can vary in time, and the distribution $p_{\textrm{constrained}}(\cdot ,t)$ remains properly normalized for each *t*.

## Dimension reduction and surface Fokker–Planck equations

The distribution $p_{\textrm{constrained}}(x,t)$ effectively lives on a $(d-A)$-dimensional manifold. One can view this as *dimension reduction* of the original problem in ${\mathbb {R}}^d$.

### Surface Fokker–Planck / Liouville equation on a moving manifold

When no constraints are imposed, the evolution of the probability density ${\widetilde{p}}(x,t)$ in the full space ${\mathbb {R}}^d$ can often be described by a standard *Fokker–Planck* (FP) or *Liouville* PDE:

$$\begin{aligned} \frac{\partial }{\partial t}\,{\widetilde{p}}(x,t) \;=\; -\nabla \cdot \Bigl [{\widetilde{p}}(x,t)\,f(x,t)\Bigr ] \;+\;\text {(diffusion terms)}, \qquad x \in {\mathbb {R}}^d. \end{aligned}$$

However, once we enforce ${\varvec{\sigma }}(x,t) = {\varvec{0}}$, the domain for *x* becomes the *moving manifold* ${\mathcal {M}}(t)\subset {\mathbb {R}}^d$ of dimension $(d-A)$. This reduction induces several significant changes to the form of the PDE governing the *constrained* density $p_{\textrm{constr}}(x,t)$:

1. The state space is now ${\mathcal {M}}(t)$ instead of the entire ${\mathbb {R}}^d$;
2. Drift and diffusion are projected *tangentially* onto ${\mathcal {M}}(t)$;
3. ${\mathcal {M}}(t)$ itself may *move* or *deform* in time, contributing a geometric (normal) flux.

*Tangential vs. Normal Directions.* Because the motion is confined to satisfy ${\varvec{\sigma }}(X(t),t)=0$, any component of the drift or diffusion pointing *normal* to ${\mathcal {M}}(t)$ is effectively removed (or canceled by Lagrange multipliers). Consequently, the probability flux is restricted to directions *tangent* to the manifold. If $v_{\textrm{tan}}(x,t)$ denotes the resulting (projected) drift/diffusion velocity *within the tangent space* of ${\mathcal {M}}(t)$, then classical vector calculus in ${\mathbb {R}}^d$ is replaced by its *surface differential geometry* analog. Specifically, one replaces the usual divergence $\nabla \cdot (\cdot )$ by the *manifold (or surface) divergence* $\nabla _{{\mathcal {M}}(t)}\cdot (\cdot )$ defined on ${\mathcal {M}}(t)$.

*Moving Domain Effects.* In addition to tangential flux, there is also a subtle effect from the motion or deformation of ${\mathcal {M}}(t)$ itself. The surface (or manifold) can move *normally* in ${\mathbb {R}}^d$ if ${\varvec{\sigma }}(x,t)$ has explicit time dependence (e.g., a bond length constraint that slowly varies in time). This motion induces an “apparent flux” of probability, even at points where the drift *along* ${\mathcal {M}}(t)$ might be zero. If $u_{\textrm{surf}}(x,t)$ denotes the local normal velocity of ${\mathcal {M}}(t)$ at $x\in {\mathcal {M}}(t)$, it contributes an extra term capturing how volume elements of ${\mathcal {M}}(t)$ expand, contract, or shift over time.

*Resulting PDE.* Collecting these two kinds of flux (tangential and normal), one obtains a *surface FP or Liouville equation* of the general form:

31 $$\begin{aligned} \frac{\partial }{\partial t}\,p_{\textrm{constr}}(x,t) \;+\; \nabla _{{\mathcal {M}}(t)}\!\cdot \Bigl [ p_{\textrm{constr}}(x,t)\,v_{\textrm{tan}}(x,t) \Bigr ] \;+\; p_{\textrm{constr}}(x,t)\;\Bigl [\nabla _{{\mathcal {M}}(t)} \cdot u_{\textrm{surf}}(x,t)\Bigr ] \;=\; 0, \end{aligned}$$

With $$\quad x\in {\mathcal {M}}(t).$$.

Here,

- $\nabla _{{\mathcal {M}}(t)}\!\cdot$ denotes the *surface divergence* operator appropriate to the manifold ${\mathcal {M}}(t)$,
- $v_{\textrm{tan}}(x,t)$ is the drift/diffusion velocity projected onto the tangent space of ${\mathcal {M}}(t)$,
- $u_{\textrm{surf}}(x,t)$ is a normal velocity indicating how the manifold *itself* is changing shape or position in time,
- the last term $\nabla _{{\mathcal {M}}(t)}\cdot u_{\textrm{surf}}(x,t)$ effectively measures how *local* changes in the manifold geometry (e.g., expansion in some directions, curvature changes) affect the probability distribution confined to that surface.

*Differences from the Original FP Equation.* Compared to the original FP equation on ${\mathbb {R}}^d$, the surface version (31)

1. *Lives entirely on* ${\mathcal {M}}(t)$: All derivatives (divergences, gradients) are restricted to directions tangent to the manifold, and the domain $x\in {\mathcal {M}}(t)$ is a lower-dimensional set that can itself vary in time.
2. *Includes an extra geometric term:* The $\nabla _{{\mathcal {M}}(t)} \cdot u_{\textrm{surf}}(x,t)$ term does not appear in the standard FP equation on fixed domains, reflecting that points *on* the manifold move in the ambient space ${\mathbb {R}}^d$ due to time-dependent constraints.
3. *Requires a surface measure or metric:* To interpret $p_{\textrm{constr}}(x,t)$ properly, one must use surface (or manifold) integration with an induced metric (and hence a Gram determinant factor in the measure).

### Interpretation: blue moon ensemble & Fixman factor

In molecular dynamics or chemical physics, restricting the system to satisfy ${\varvec{\sigma }}(x)=0$ (such as bond-length constraints) leads to what is sometimes called the “Blue Moon ensemble” [36]. The manifold ${\mathcal {M}}\subset {\mathbb {R}}^d$ is a reduced-dimensional configuration space, and a correction factor (the determinant of $\nabla {\varvec{\sigma }}\,\nabla {\varvec{\sigma }}^\top$) must be included when mapping the ambient measure to ${\mathcal {M}}$. For time-*independent* constraints in equilibrium, this becomes the *Fixman* correction to the Boltzmann factor [37]. Our Theorem 3 extends that logic to *time-varying* constraints ${\varvec{\sigma }}(x,t)$, ensuring a properly normalized measure on the moving manifold.

## KL divergence constraint

It may be of interest to reformulate the constraint optimization problem in the form of minimizing the KL divergence over constraint distributions. This has advantages because assigning constraints to specific batch elements at inference is no longer necessary. Instead, we can talk about how distributions of constraints are satisfied instead of individual constraints. To incorporate distance constraints during diffusion processes, we iteratively adjust atomic positions to minimize deviations from the target constraints. The algorithm updates the positions $x_i$ and $x_j$ of the atoms involved in the constraint using the following steps.

### KL divergence constraint

Given the positions of atoms ${\textbf{x}}_i^{(t)}$ and ${\textbf{x}}_j^{(t)}$ at the $t$-th diffusion step, the distance between them is:

32 $$\begin{aligned} d_{ij}^{(t)} = \Vert {\textbf{x}}_i^{(t)} - {\textbf{x}}_j^{(t)} \Vert \end{aligned}$$

Assume that the target distribution $P(d)$ and the current distribution $Q(d)$ are Gaussian distributions:

$$\begin{aligned} P(d) = {\mathcal {N}}(\mu _P, \sigma _P^2) \\ Q(d) = {\mathcal {N}}(\mu _Q, \sigma _Q^2) \end{aligned}$$

The KL divergence between two Gaussian distributions $P$ and $Q$ is given by:

33 $$\begin{aligned} D_{KL}(P \Vert Q) = \log \left( \frac{\sigma _Q}{\sigma _P} \right) + \frac{\sigma _P^2 + (\mu _P - \mu _Q)^2}{2\sigma _Q^2} - \frac{1}{2} \end{aligned}$$

### Gradient calculation

The gradients of the KL divergence with respect to the parameters $\mu _Q$ and $\sigma _Q^2$ are:

34 $$\begin{aligned} \frac{\partial D_{KL}(P \Vert Q)}{\partial \mu _Q}= & \frac{\mu _Q - \mu _P}{\sigma _Q^2} \end{aligned}$$

35 $$\begin{aligned} \frac{\partial D_{KL}(P \Vert Q)}{\partial \sigma _Q^2}= & \frac{1}{2} \left( \frac{\sigma _P^2 + (\mu _P - \mu _Q)^2}{\sigma _Q^4} - \frac{1}{\sigma _Q^2} \right) \end{aligned}$$

Assume that $\mu _Q$ and $\sigma _Q^2$ depend on the distances $d_{ij}$:

$$\begin{aligned} \mu _Q= & f(\{ d_{ij} \}) \\ \sigma _Q^2= & g(\{ d_{ij} \}) \end{aligned}$$

The gradients of $D_{KL}(P \Vert Q)$ with respect to $d_{ij}$ are then given by:

36 $$\begin{aligned} \frac{\partial D_{KL}(P \Vert Q)}{\partial d_{ij}} = \frac{\partial D_{KL}(P \Vert Q)}{\partial \mu _Q} \cdot \frac{\partial \mu _Q}{\partial d_{ij}} + \frac{\partial D_{KL}(P \Vert Q)}{\partial \sigma _Q^2} \cdot \frac{\partial \sigma _Q^2}{\partial d_{ij}} \end{aligned}$$

Since $d_{ij} = \Vert {\textbf{x}}_i - {\textbf{x}}_j \Vert$, we have:

37 $$\begin{aligned} \frac{\partial d_{ij}}{\partial {\textbf{x}}_i}= & \frac{{\textbf{x}}_i - {\textbf{x}}_j}{\Vert {\textbf{x}}_i - {\textbf{x}}_j \Vert } = \frac{{\textbf{x}}_i - {\textbf{x}}_j}{d_{ij}} \end{aligned}$$

38 $$\begin{aligned} \frac{\partial d_{ij}}{\partial {\textbf{x}}_j}= & \frac{{\textbf{x}}_j - {\textbf{x}}_i}{\Vert {\textbf{x}}_j - {\textbf{x}}_i \Vert } = \frac{{\textbf{x}}_j - {\textbf{x}}_i}{d_{ij}} \end{aligned}$$

Therefore:

39 $$\begin{aligned} \frac{\partial D_{KL}(P \Vert Q)}{\partial {\textbf{x}}_i}= & \left( \frac{\mu _Q - \mu _P}{\sigma _Q^2} \cdot \frac{\partial \mu _Q}{\partial d_{ij}} + \frac{1}{2} \left( \frac{\sigma _P^2 + (\mu _P - \mu _Q)^2}{\sigma _Q^4} - \frac{1}{\sigma _Q^2} \right) \cdot \frac{\partial \sigma _Q^2}{\partial d_{ij}} \right) \cdot \frac{{\textbf{x}}_i - {\textbf{x}}_j}{d_{ij}} \end{aligned}$$

40 $$\begin{aligned} \frac{\partial D_{KL}(P \Vert Q)}{\partial {\textbf{x}}_j}= & \left( \frac{\mu _Q - \mu _P}{\sigma _Q^2} \cdot \frac{\partial \mu _Q}{\partial d_{ij}} + \frac{1}{2} \left( \frac{\sigma _P^2 + (\mu _P - \mu _Q)^2}{\sigma _Q^4} - \frac{1}{\sigma _Q^2} \right) \cdot \frac{\partial \sigma _Q^2}{\partial d_{ij}} \right) \cdot \frac{{\textbf{x}}_j - {\textbf{x}}_i}{d_{ij}} \end{aligned}$$

These gradients can be used to iteratively adjust the positions ${\textbf{x}}_i$ and ${\textbf{x}}_j$ to minimize the KL divergence, ensuring that the distances conform to the target distribution $P(d)$.

In molecular generative modeling, a key challenge is to ensure that generated samples exhibit certain geometric properties reflective of realistic molecular structures. Traditional approaches typically impose constraints directly on individual samples, specifying which elements must satisfy which constraints. This can become both computationally and combinatorially expensive, especially when dealing with large batches or numerous constraints.

A more scalable approach is to enforce these constraints *distributionally*. Rather than requiring every generated configuration to adhere exactly to specified geometric constraints, we dictate that the entire *distribution* of geometric features (e.g., bond lengths, angles, torsions) in the generated set match a given *target distribution* derived from empirical data or theoretical models.

### Distributional constraints and induced distributions

Let ${\mathcal {X}}$ denote the space of molecular conformations (e.g., sets of 3D coordinates of atoms), and let $Z: {\mathcal {X}} \rightarrow {\mathbb {R}}$ represent a geometric feature of interest, such as a particular bond length or angle. Suppose we have a target distribution $P_Z$ characterizing the desired statistical profile of *Z*. For instance, $P_Z$ might be a Gaussian with mean and variance determined from a reference database of experimentally validated molecules.

A generative model *G* induces a distribution $Q_Z$ over *Z* by producing samples $x \sim G$ and then mapping these samples to their corresponding *Z*(*x*) values. The fundamental objective is to ensure $Q_Z$ aligns with $P_Z$.

### KL divergence as a measure of alignment

The KL divergence between $P_Z$ and $Q_Z$ is defined as:

41 $$\begin{aligned} D_{KL}(P_Z \Vert Q_Z) \;=\; \int P_Z(z) \log \frac{P_Z(z)}{Q_Z(z)}\,dz. \end{aligned}$$

This quantity satisfies $D_{KL}(P_Z \Vert Q_Z) \ge 0$, with equality if and only if $P_Z$ and $Q_Z$ coincide on the support of $P_Z$. More precisely:

#### Theorem 5

(Equivalence of Zero KL Divergence and Distributional Equality) Suppose $P_Z$ and $Q_Z$ are probability distributions on ${\mathbb {R}}$ such that $P_Z$ is absolutely continuous with respect to some base measure $\mu$ (e.g., the Lebesgue measure) and $Q_Z$ is absolutely continuous with respect to $\mu$ as well. Then:

$$\begin{aligned} D_{KL}(P_Z \Vert Q_Z) = 0 \quad \iff \quad P_Z(z)=Q_Z(z) \text { for } \mu \text {-almost all } z \text { where } P_Z(z)>0. \end{aligned}$$

In other words, if there is any set of *z* with positive $P_Z$-measure on which $P_Z(z) \ne Q_Z(z)$, then $D_{KL}(P_Z \Vert Q_Z) > 0$.

#### Proof

If $$D_{KL}(P_Z \Vert Q_Z)=0$$, then

$$\begin{aligned} \int P_Z(z)\log \frac{P_Z(z)}{Q_Z(z)}\,dz = 0. \end{aligned}$$

Since $\log \frac{P_Z(z)}{Q_Z(z)} \ge 0$ for all *z* where $Q_Z(z)>0$, the integrand must be zero almost everywhere with respect to the measure induced by $P_Z$. Thus, for $P_Z$-almost every *z*, $\log \frac{P_Z(z)}{Q_Z(z)}=0$, which implies $P_Z(z)=Q_Z(z)$. Conversely, if $P_Z(z)=Q_Z(z)$ for $P_Z$-almost every *z*, the integrand is zero on that set and $D_{KL}(P_Z \Vert Q_Z)=0$. $\square$

Note that the phrase *“for *$P_Z$-*almost every* *z*” is a precise measure-theoretic statement, meaning the set of *z* where $P_Z(z)$ differs from $Q_Z(z)$ has $P_Z$-measure zero. This guarantees that $P_Z$ and $Q_Z$ represent the same probability law. If both distributions are given by probability density functions (pdfs) with respect to a common measure $\mu$, then $P_Z=Q_Z$ $\mu$-almost everywhere on the support of $P_Z$.

### Multiple geometric constraints

In general, we may have multiple geometric features $Z_1, Z_2, \ldots , Z_K$, each with a target distribution $P_{Z_k}$ and an induced distribution $Q_{Z_k}$. We aggregate these divergences:

42 $$\begin{aligned} {\mathcal {L}}_{KL} \;=\; \sum _{k=1}^K D_{KL}(P_{Z_k} \Vert Q_{Z_k}). \end{aligned}$$

Since each term is non-negative, ${\mathcal {L}}_{KL}=0$ if and only if $D_{KL}(P_{Z_k}\Vert Q_{Z_k})=0$ for all *k*. By Theorem 5, this means $P_{Z_k}=Q_{Z_k}$ for each *k*, ensuring that *all* geometric features match their target distributions.
